# Novel family of bis-pyrazole coordination complexes as potent antibacterial and antifungal agents†

**DOI:** 10.1039/d2ra03414j

**Published:** 2022-06-15

**Authors:** Youssef Draoui, Smaail Radi, Amine Tanan, Afaf Oulmidi, Haralampos N. Miras, Redouane Benabbes, Sabir Ouahhoudo, Samira Mamri, Aurelian Rotaru, Yann Garcia

**Affiliations:** LCAE, Department of Chemistry, Faculty of Science, University Mohamed I P.O. Box 524 Oujda 60 000 Morocco s.radi@ump.ac.ma; Institute of Condensed Matter and Nanosciences, Molecular Chemistry, Materials and Catalysis (IMCN/MOST), Université Catholique de Louvain Belgium yann.garcia@uclouvain.be +32-10472330; School of Chemistry, Joseph Black Building, University of Glasgow Glasgow G12 8QQ UK; Laboratory of Biochemistry and Biotechnology, Department of Biology, Faculty of Science, University Mohamed I P.O. Box 524 Oujda 60 000 Morocco; Department of Electrical Engineering and Computer Science & Research Center MANSiD, “Stefan Cel Mare” University University Street, No. 13 Suceava 720229 Romania

## Abstract

A new pyrazole ligand, *N*,*N*-bis(2(1′,5,5′-trimethyl-1*H*,1′*H*-[3,3′-bipyrazol]-1-yl)ethyl)propan-1-amine (L) was synthesized and characterized by ^1^H-NMR, ^13^C-NMR, FT-IR and HRMS. The coordination ability of the ligand has been employed for the construction of a new family of coordination complexes, namely: [Cu_2_LCl_4_] (1), [ML(CH_3_OH)(H_2_O)](ClO_4_)_2_ (M^II^ = Ni (2), Co (3)) and [FeL(NCS)_2_] (4). The series of complexes were characterized using single-crystal X-ray diffraction, HRMS, FT-IR and UV-visible spectroscopy. Moreover, the iron(ii) analogue was investigated by ^57^Fe Mössbauer spectroscopy and SQUID magnetometry. Single-crystal X-ray structures of the prepared complexes are debated within the framework of the cooperative effect of the hydrogen bonding network and counter anions on the supramolecular formations observed. Furthermore, within the context of biological activity surveys, these compounds were reviewed against different types of bacteria to validate their efficiency, including both Gram-positive as well as Gram-negative bacteria. Enhanced behaviour towards *Fusarium oxysporum* f. sp. *albedinis* fungi, were detected for 1 and 4.

## Abstract 2

A new pyrazole ligand L and four coordination complexes were synthesized and characterized by different spectroscopic methods. These were found to be promising antibacterial and antifungal agents.

## Graphical abstract

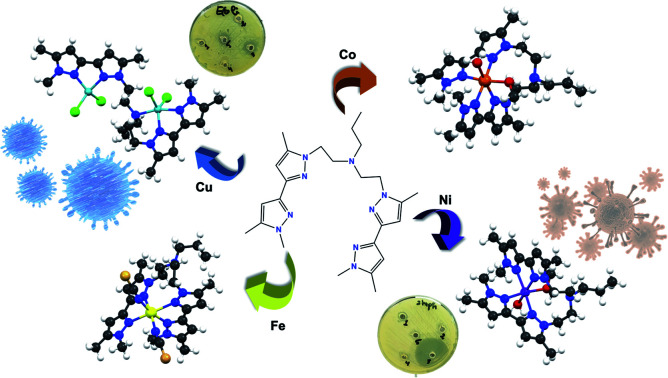

## 1. Introduction

Nitrogen heterocyclic containing molecules play an important role in the recognized structural diversity observed in the coordination chemistry of these ligands with metal ions. More specifically, pyrazole-based ligands have proven their efficiency for the construction of coordination complexes with useful applications in several fields not only in catalysis,^1^ agricultural biotechnology,^2^ corrosion inhibition, antimicrobial activity^3^ and biology,^4^ but also in pharmaceutical and medical research,^5^ leading for instance to anti-inflammatory^6,7^ and anti-cancer agents.^8^

According to recent literature reports,^9,10^ the preparation of pyrazole based ligands with multiple coordination sites remains a challenging task. Such five-membered heterocyclic ligands associated with hydrogen bonding as well as π–π interactions can lead to interesting features, such as flexibility, ductility and chelation properties for the resulting coordination complexes.^11^ The construction of these complexes depends on the effect of hydrogen bonding,^12^ donor atoms,^13^ counter anions,^14^ solvent^15^ and the topologies of the selected ligand.^16^ In addition, such organic molecules were found to exhibit various properties, be pharmacological,^17^ chemosensory and photophysical,^18^ optical and morphological properties,^19^ to name but a few. Thus, coordination complexes formed with pyrazole building blocks may offer excellent compounds for the exploration of new functionalities and investigation of chemical and electronic behaviours. For example, a large number of pyrazole-based coordination complexes with cobalt and copper revealed interesting antioxidant activity.^20^ Moreover, pyrazole iron complexes, revealed remarkable electronic properties (*e.g.* spin crossover phenomena^21,22^) or catalytic selectivity (in the hydrosilylation of organocarbonyl substrates^23^). Additionally, relevant pyrazolate complexes of cadmium, nickel and mercury were found to act as efficient antimicrobial agents due to the induced disruption of the cell membrane's integrity in the case of Gram positive and Gram negative bacteria.^24^ Recent examples of iridium and platinum pyrazolate complexes demonstrated too highly promising activity in medical applications as in the case of anti-cancer agents.^25,26^

Inspired by the observed structural diversity and wide range of applications, our group developed new pyrazolate derivatives^27,28^ and investigated their efficiency in the coordination of transition metals. This research objective is actually the first step in the construction of environmentally friendly hybrid silica-based materials for extraction of transition metals and purification of industrial waste water.^29,30^

**Scheme 1:**
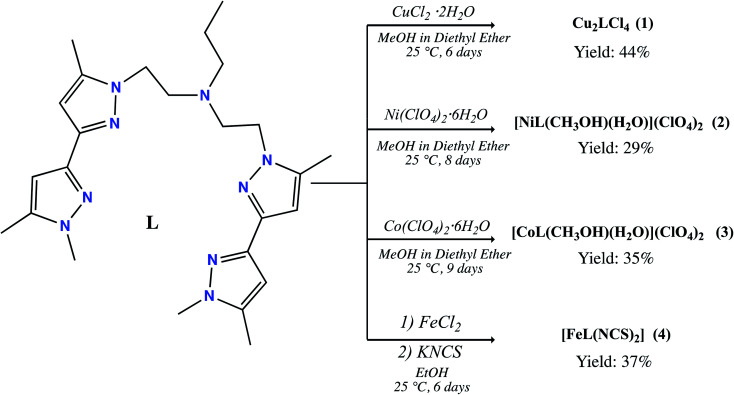
Synthesis of bis-pyrazole coordination complexes 1–4.

In this work, we discuss the preparation and characterisation of a new highly flexible pyrazole ligand *N*,*N*-bis(2(1′,5,5′-trimethyl-1*H*,1′*H*-[3,3′-bipyrazol]-1-yl)ethyl)propan-1-amine (L) which exhibits multiple coordination sites due to the presence of four pyrazolate rings. It was characterised by ^1^H NMR, ^13^C NMR, infra-red spectroscopy, and high-resolution mass spectrometry. Moreover, the ligand was used for the synthesis of four new coordination complexes (Scheme 1), namely: [Cu_2_LCl_4_] (1), [ML(CH_3_OH)(H_2_O)] (M^II^ = Ni (2), Co (3)) and [FeL(NCS)_2_] (4). The complexes were characterised by single crystal X-ray diffraction, elemental analysis, FT-IR and UV-visible spectroscopy. Additionally, the electronic structure of the iron pyrazolate adduct was investigated further using ^57^Fe Mössbauer spectroscopy and SQUID magnetometry. The antibacterial properties of L and 1–4 were investigated against *Staphylococcus aureus* and *Streptococcus* spp as Gram-positive specimens also *Escherichia coli* and *Klebsiella* spp. as Gram-negative specimens. Their antifungal performance *versus Fusarium oxysporum* f.sp. *albedinis* fungi was also reviewed.

## 2. Experimental section

### 2.1. Materials and instrumentation

All solvents and chemicals, obtained from usual commercial sources, were of analytical grade and used without further purification. ^1^H and ^13^C NMR spectra were obtained from a Bruker AC 300 spectrometer. High resolution mass spectrometry HRMS data were obtained with a Q Exactive Thermofisher Scientific ion trap spectrometer by using ESI ionization. FT-IR spectra were recorded on potassium bromide discs using a PerkinElmer 1310 spectrometer. UV-visible spectra were recorder using a Shimadzu 3600 plus spectrometer equipped with Harrick praying mantis modulus which allows direct analysis of powders in reflectance mode. Melting points were measured using a Koffler bench. Magnetic susceptibilities were measured on a Quantum design MPMS3 SQUID magnetometer. Magnetic data were corrected for the sample holder and diamagnetic contributions. A polycrystalline sample of 4 was quickly loaded into a gelatine capsule and immediately inserted within the SQUID cavity to avoid any air oxidation. ^57^Fe Mössbauer spectra were recorded in transmission geometry with a constant acceleration mode conventional spectrometer equipped with a 50 mCi ^57^Co(Rh) source and a Reuter Stokes proportional counter. The powdered sample was sealed in plastic sample holder and a spectrum was recorded at 298 K. The spectrum was fitted using Recoil 1.0 Mössbauer analysis software^31^). The isomer shift values are given with respect to α-Fe at room temperature.

### 2.2. X-ray crystallography

Suitable single crystals were selected and mounted onto a rubber loop using Fomblin oil. Single-crystal X-ray diffraction data of 1–4 were recorded on a Bruker Apex CCD diffractometer (*λ* (Mo K_α_) = 0.71073 Å) at 150 K equipped with a graphite monochromator. Structure solution and refinement were carried out with SHELXS-97 ^32^ and SHELXL-97 ^33^ using the WinGX software package.^34^ Data collection and reduction were performed using the Apex2 software package. Corrections for incident and diffracted beam absorption effects were applied using empirical absorption corrections.^35^ All the atoms and most of the carbon atoms were refined anisotropically. Solvent molecule sites were found and included in the refinement of the structures. Final unit cell data and refinement statistics for 1–4 are collected in Table S1.† Crystallographic data for compounds 1–4 (CCDC 1: 2154263, 2: 2154264, 3: 2154265 and 4: 2154266).†

### 2.3. Synthesis section

The pyrazole derivatives from A1 to A6 were prepared and characterized according to our previous procedure.^36^

#### 2.3.1. Synthesis of *N*,*N*-bis(2-(1′,5,5′-trimethyl-1*H*,1′*H*-[3,3′-bipyrazol]-1-yl)ethyl) propan-1-amine (L)

To a solution of 2-(1′,5,5′-trimethyl-1*H*,1′*H*-[3,3′-bipyrazol]-1-yl)ethan-1-ol (A6) (4 g, 18.1 mmol, 1 equiv.) in dichloromethane (80 mL), *p*-toluenesulfonyl chloride (4.15 g, 21.8 mmol, 1.2 equiv.) was added and the mixture was cooled to 0 °C. To the above solution, NaOH (1.45 g, 3.6 mmol, 2 equiv.) was added and the mixture was stirred for 5 h at the same temperature. Water (30 mL) was added to the resulting mixture, then extracted. This process was repeated three times. The organic phase was dried with sodium sulphate and the product was obtained as a white powder after the solvent was reduced under vacuum. Yield 60% (4.1 g).

The tosylated product (4.1 g, 10.9 mmol, 2 equiv.) was dissolved in 80 mL of acetonitrile then potassium carbonate (4.39 g, 32.8 mmol, 3 equiv.) was added. To the resulting mixture, propylamine (0.32 g, 5.5 mmol, 1 equiv.) dissolved preliminary in acetonitrile (10 mL), was added dropwise and the reaction mixture was refluxed for 15 days (82 °C) in a closed vessel. After filtration, the solvent was halfway evaporated under vacuum, then the mixture was left in the fridge at 0 °C for 12 hours. A white precipitate was formed, after filtration, the white powder was washed with cold acetonitrile (5 mL) and then diethyl ether (10 mL). Yield 30% (1.5 g). M.p 188 °C. ^1^H-NMR (300 MHz, DMSO-d6) *δ* ppm: 0.81 (t, *J* = 7.4 Hz, 3H, CH_3_–CH_2_), 1.41 (q, *J* = 7.4 Hz 2H, CH_3_–CH_2_–CH_2_), 2.24 (s, 3H, CH_3_–C), 2.27 (s, 3H, CH_3_–C), 2.45 (t, *J* = 7.4 Hz, 2H, Niso–CH_2_–CH_2_), 2.91 (t, *J* = 6.8 Hz 2H, CH_2_–CH_2_-Nprop), 3.79 (s, 3H, CH_3_–N), 3.97 (t, *J* = 6.8 Hz 2H, NPz–CH_2_–CH_2_), 6.24 (s, 1H, C–CH–C), 6.28 (s, 1H, C–CH–C). ^13^C-NMR (75 MHz, DMSO-d6) *δ* ppm: 11.3 (s, 2C, CH_3_–C, Pz), 11.8 (s, 2C, CH_3_–C, Pz-tripod), 20.6 (s, 1C, CH_3_–CH_2_), 31.0 (s, 1C, CH_3_–CH_2_–CH_2_), 36.1 (s, 2C, CH_3_–N), 47.8 (s, 2C, CH_3_–NPz), 55.0 (s, 2C, CH_2_–CH_2_–Nprop), 57.4 (s, 1C, CH_2_–CH_2_–Niso), 102.7 (s, 2C, C–C=C, Pz), 102.9 (s, 2C, C–C=C, Pz-tripod) 139.2 (s, 2C N–C, Pz) 139.4 (s, 2C, N–C, Pz-tripod) 145.1 (s, 2C, C–C–Pz), 145.3 (s, 2C, C–C, Pz-tripod). FTIR (KBr, cm^−1^): 3143 (w), 2969 (w), 1537 (s), 1422 (m), 1230 (m), 929 (w), 780 (m). HRSM (ESI) (methanol): *m*/*z* = 464.3244 [M + H]^+^.

#### 2.3.2. Synthesis of Cu_2_LCl_4_ (1)

Ligand (L) (45 mg, 0.097 mmol, 1 equiv.) was dissolved in methanol (4 mL). CuCl_2_·2H_2_O (33.2 mg, 0.195 mmol, 2 equiv.) was dissolved in methanol (3 mL) and added to the previous solution of (L), and stirred for 10 min at room temperature. The resulting mixture was allowed to vapor diffuse with diethyl ether (10 mL). Dark green single crystals were obtained after 6 days, washed with a mixture of diethyl ether and methanol (8 : 2), and collected. Yield 44% (31 mg). M.p 208 °C. FT-IR (KBr, cm^−1^): 3129 (w), 2955 (w), 1544 (s), 1425 (m), 1276 (m), 945 (w), 792 (m). HRMS (ESI) (acetonitrile): *m*/*z* = 561.2145 [L + CuCl]^+^, 659.11254 [L + Cu_2_Cl_2_]^+^, 263.12237 [1/2(L + Cu)]^+^.

#### 2.3.3. Synthesis of [NiL(CH_3_OH)(H_2_O)](ClO_4_)_2_ (2)

Ligand (L) (45 mg, 0.097 mmol, 1 equiv.) was dissolved in methanol (4 mL). Ni(ClO_4_)_2_·6H_2_O (35.8 mg, 0.098 mmol, 1 equiv.) was dissolved in methanol (3 mL) and added to the previous solution of (L), and stirred for 10 min at room temperature. The resulting mixture was allowed to vapor diffuse with diethyl ether (10 mL). Blue single crystals were obtained after 8 days, washed with a mixture of diethyl ether and methanol (8 : 2), and collected. Yield 29% (21 mg). M.p 200 °C. FTIR (KBr, cm^−1^): 3132(w), 2960(w), 1546(s), 1416(m), 1233(m), 943(w), 784(m). HRMS (ESI) (acetonitrile): *m*/*z* = 260.6259 [1/2(L + Ni)]^+^.

#### 2.3.4. Synthesis [CoL(CH_3_OH)(H_2_O)](ClO_4_)_2_ (3)

Ligand (L) (45 mg, 0.097 mmol, 1 equiv.) was dissolved in methanol (4 mL). Co(ClO_4_)_2_·6H_2_O (36.5 mg, 0.1 mmol, 1 equiv.) was dissolved in methanol (3 mL) and added to the previous solution of (L), and stirred for 10 min at room temperature. The resulting mixture was allowed to vapor diffuse with diethyl ether (10 mL). Orange single crystals were obtained after 9 days, washed with a mixture of diethyl ether and methanol (8 : 2), and collected. Yield 35% (26 mg). M.p. 192 °C. FT-IR (KBr, cm^−1^): 3131(w), 2963(w), 1554(s), 1430(m), 1278(m), 947(w), 801(m). HRSM (ESI) (acetonitrile): *m*/*z* = 261.1246 [1/2(L + Co)]^+^.

#### 2.3.5. Synthesis of [FeL(NCS)_2_] (4)

An ethanolic solution (2 mL) containing (19.5 mg, 0.098 mmol, 1 equiv.) of FeCl_2_·4H_2_O mixed with a small amount of ascorbic acid (5 mg) was rapidly added to a hot ethanol solution (2 mL) of KNCS (19 mg, 0.196 mmol, 2 equiv.). The mixture was added dropwise to an ethanolic solution (5 mL) of L (45 mg, 0.097 mmol, 1 equiv.). The resulting solution was left at room temperature to lead to yellow single crystals after a period of 6 days of slow evaporation, washed with ethanol and collected. Yield 37% (22 mg). M.p. 230 °C. FT-IR (KBr, cm^−1^): 3133(w), 2961(w), 2066(s), 1541(s), 1428(m), 1237(m), 935(w), 786(m). HRMS (ESI) (acetonitrile): *m*/*z* = 575.2326 [L + H_2_FeNCS]^+^.

### 2.4. Antibacterial activities

Antibacterial activities were evaluated using the disk diffusion method on agar with some modifications.^37,38^ Fifteen milliliters of Mueller–Hinton agar (45 °C) were poured into sterile Petri dishes (*ϕ* 90 mm). Each organism was separately suspended in a normal saline solution and transmittance (*T*) of 75–77% at *λ* = 530 nm was made, which is equal to 10^6^ CFU mL^−1^. Each bacterial suspension was uniformly spread on a solid growth medium in a Petri dish. Once the plates were aseptically dried, 6 mm holes were drilled into the agar using a sterile Pasteur pipette. 100 μL of a previously prepared solution containing each compound (4 mg mL^−1^) was placed in the holes. The plates were incubated at 37 °C for 24 h. DMSO was used as a negative control since it has no antibacterial activities. On the other hand, lactic acid was used as a positive control due to its decent behavior against various bacteria types, while the antibiotic gentamycin was set to refer to the better positive control. The positive antimicrobial activity was read based on growth inhibition zone and compared with the solvent, as control. The tests were performed in triplicate, the average value of the three are reported.

### 2.5. Antifungal activities

#### 2.5.1. Preparation of the substances

The preparation of the stock solution was performed by solubilizing 4 mg of each compound with DMSO (1 mL).

#### 2.5.2. Preparation of the fungus

First, the FOA was isolated from Boufegousgharas date palm from Figuig in Morocco that is infected by the vascular fusariosis according to a literature protocol.^39^

#### 2.5.3. Antifungal activities protocol

Distilled water (1 L) was added to PDA (39 g). The mixture was refluxed for 10 min, then sterilized at 120 °C for 20 min. After cooling, 3 sterile tubes were filled with respective volumes (50 μL, 100 μL and 200 μL) and completed up to 15 mL with liquid PDA, then spread over 8.5 cm diameter Petri dishes and then left at room temperature until solidification.^40^ After that, cultivated FOA was transplanted onto a pellet in the center of each Petri dish. The prepared Petri dishes were incubated at 28 °C for 5 days. The results are expressed as % inhibition by measuring the diameter of the FOA compared to the negative control containing only PDA, FOA and DMSO (200 μL).^41^ The tests were performed in triplicate; the average value of the three are reported.
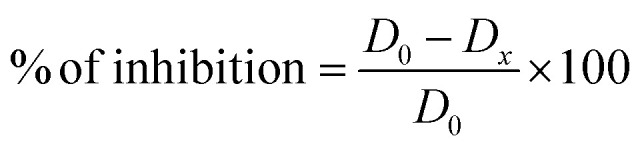
*D*_0_: diameter (cm) of FOA in the control, *D*_*x*_: diameter (cm) of FOA in the test.

## 3. Result and discussion

### 3.1. Synthesis and characterization

A large number of pyrazole based complexes have been synthesized over the last decades.^42,43^ Our objective was to increase the number of coordination sites and flexibility of such ligands by designing a new ligand made of a bis-pyrazole synthon, namely 1,5,5′-trimethyl-1*H*,1′*H*-3,3′-bipyrazole. The synthesis was undertaken following a simple reaction pathway. Indeed, the reaction of *p*-toluenesulfonyl chloride with 2-(1′,5,5′-trimethyl-1*H*,1′*H*-[3,3′-bipyrazol]-1-yl)ethan-1-ol in the presence of sodium hydroxide in dichloromethane led to the tosyl-based derivative (yield 60%). Our envisaged target was a symmetric molecule with a double bis-pyrazole, thus our ligand design plan involved an alkylation of the present tosylated product to a propylamine in acetonitrile. The desired product L was obtained easily in the presence of a base as white powder due to the easy leaving group properties of the tosyl group.

Reaction of the ligand (L) with different metal salts, in methanol in 1 : 2 or 1 : 1 molar ratio was carried out. Reaction with CuCl_2_·2H_2_O in a 1 : 2 molar ratio afforded a binuclear Cu(ii) complex (1) which was crystallized by diethyl ether vapour diffusion. Reaction with Ni(ClO_4_)_2_·6H_2_O or Co(ClO_4_)_2_·6H_2_O salts in a 1 : 1 molar ratio yielded to a mononuclear Ni(ii) complex (2) which was also crystallized by diethyl ether vapour diffusion. The complex 4 was however synthetized in two steps involving the reaction of FeCl_2_·4H_2_O with KNCS in 1 : 2 molar ratio in ethanol with a small quantity of ascorbic acid to prevent oxidation of the metal centre. This process increased the reactivity of the iron salt and avoided the involvement of chloride anions during the coordination reaction with the ligand. In a second step, L was reacted with freshly prepared Fe(NCS)_2_ in ethanol in a 1 : 1 molar ratio to yield to an Fe(ii) complex (4). Single crystals were obtained by slow evaporation in ethanol.

### 3.2. FT-IR and diffuse reflectance UV-visible spectroscopy

The ligand (L) exhibits some characteristic IR bands, centred at 3143, 2960, 1537 and 1230 cm^−1^ which can be assigned to the pyrazole N–H, the C–H aromatic vibrations, the aromatic C=C and the aliphatic tertiary amine, respectively (Fig. S1†). Noticeable shifts in the wavenumber were observed after coordination. Indeed, in the case of complexes 1–4, the stretching vibration associated to the N–H group at 3143 cm^−1^ was shifted to 3129 cm^−1^ (1), 3132 cm^−1^ (2), 3131 cm^−1^ (3) and 3133 cm^−1^ (4). On the other hand, the peaks associated with the C=C aromatic rings vibration centred at 1537 cm^−1^ was shifted to 1544 cm^−1^ (1), 1546 cm^−1^ (2), 1554 cm^−1^ (3) and 1541 cm^−1^ (4). The C–H aromatic vibrations observed at 2960 cm^−1^ for L have shifted to 2955, 2964, 2963 and 2961 upon complexation in 1, 2, 3 and 4, respectively. Furthermore, the aliphatic tertiary amine respectively noticed at 1230 cm^−1^ also shifted to 1278, 1277, 1280, and 1275 cm^−1^ respectively for 1, 2, 3 and 4. Non coordinated perchlorate anions were also detected at 1084 and 1093 cm^−1^ for 2 and 3, respectively. A strong band was observed at 2066 cm^−1^ for 4, corresponding to the axial NCS ligand.

The electronic spectra of L and the complexes 1–4 were recorded by diffuse reflectance spectroscopy on solids. Absorption bands in the UV region (∼200–300 nm) are observed, which are assigned to intra-ligand transitions such as π–π* and n–π*, followed by a band around 320 nm assigned to metal-to-ligand charge transfer (MLCT) processes (Fig. S2†). Complex 1 reveals in addition a large band centred around 525 nm and a well-shaped band centred at *λ*_max_ = 760 nm. These two bands may be attributed to the two chromophores revealed in 1 by single crystals-X-ray diffraction (see next section). Complexes 2 reveals too a band centred at *λ*_max_ = 610 nm of moderate intensity corresponding to d–d transitions whereas a weak band is found at *λ*_max_ = 445 nm for 3.

### 3.3. Structural characterization

**Fig. 1:**
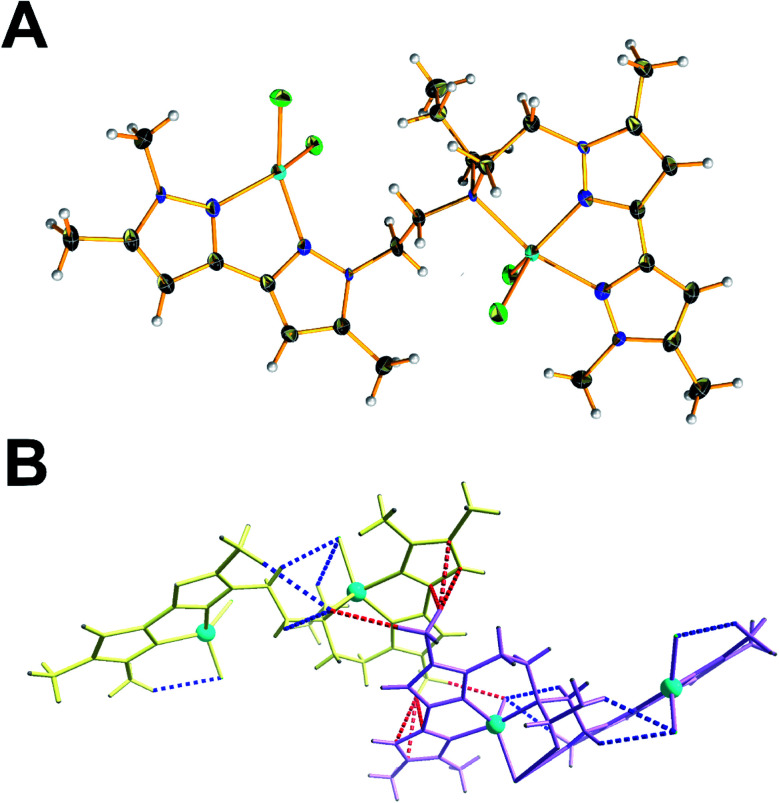
(A) Ball and stick representation of 1. Colour code: C, black; N, nitrogen; green, chloride; light grey, hydrogen; cyan, copper. Co-crystallised solvent molecules have been omitted for clarity. (B) Wire stick representation for two neighbouring molecular species of 1. Their close contact interactions are highlighted with dotted lines. Colour code: blue dotted line, intermolecular; red-dotted line, intramolecular.

All compounds were crystallised from alcoholic solutions either by vapour diffusion of diethyl ether into methanolic mother liquors (1, 2 and 4) or by slow evaporation of the solvent (3). Compound 1 crystalized in the triclinic *P̄*1 space group. The unit cell contains two molecular species and two co-crystallized methanol molecules. The two distinct copper centres are in the second oxidation state and occupy the centre of a distorted tetrahedral and square pyramidal coordination spheres which are completed by two terminal chloride ligands and two nitrogen atoms provided by the two branches of organic ligand in the case of Cu1 and two terminal chloride ligands and three nitrogen atoms in the case of Cu2, respectively (Fig. 1A). The average Cu–Cl bond lengths are found to be between 2.206(1)–2.3762(8) Å while the distances between the Cu(ii) metal centres and the pyrazolic nitrogen atoms fall in the range 2.013(2)–2.052(2) Å, respectively. The N–N bond distances are in the range of 1.348(2)–1.363(2) Å, which is characteristic for pyrazole. The unit cell contains two molecular species where the presence of inter- and intramolecular interactions contribute to the stability of the crystal packing. More specifically, the intermolecular interactions (2.701(2) Å) highlighted in blue (Fig. 1B) are developed between the terminal chloro-ligands and the hydrogen atoms of the pyrazolic ring's methyl-groups. In a similar manner, a second set of intramolecular interactions are highlighted in red dotted lines and are developed between the methyl groups of one dimer with the pyrazolic ring of the neighbouring one (Fig. 1B).

**Fig. 2:**
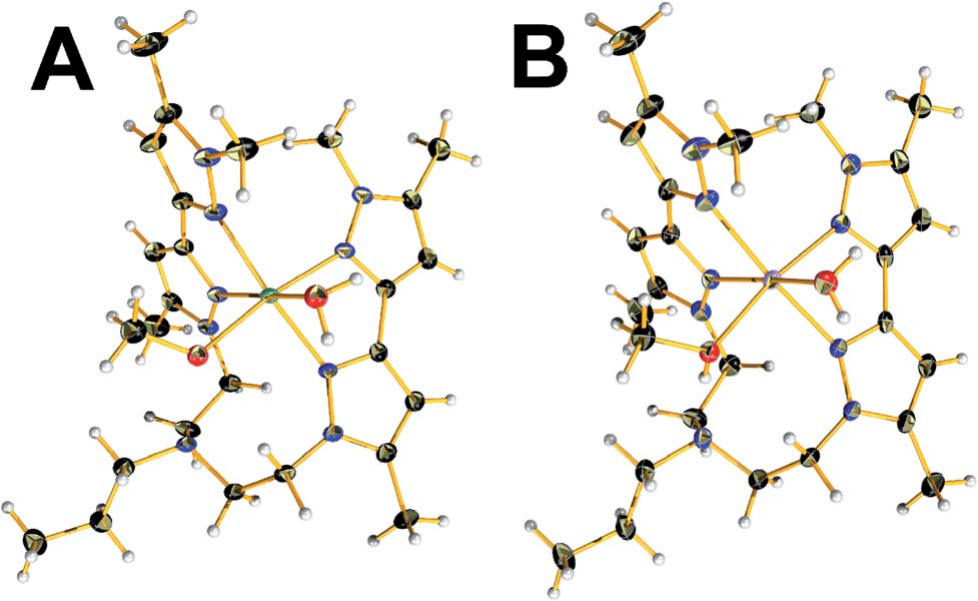
Ball and stick representation of [ML(CH_3_OH)(H_2_O)](ClO_4_)_2_ with M = Ni (A, 2) and M = Co (B, 3). Colour code: C, black; N, nitrogen; red, oxygen; light grey, hydrogen; green, nickel; pink, cobalt. Co-crystallised solvent molecules and counterions have been omitted for clarity.

Compound 2 crystalized in the monoclinic *P*2_1_/*c* space group. The unit cell contains four molecular species and two co-crystallized perchlorate counterions per complex. The Ni(ii) centre is in the second oxidation state and occupy the centre of a distorted octahedral coordination sphere which is completed by four nitrogen pyrazolic atoms provided by the two branches of L, one water and a methanol solvent molecule, respectively (Fig. 2). Interestingly, the geometry deviates from the one observed in the case of 1, while the ligand coordinates to the metal centre only *via* pyrazolic nitrogen atoms. The Ni–O bond lengths observed in the water and methanol terminal ligands are found to be 2.099(1) and 2.092(1) Å while the distances between the Ni(ii) metal centre and the pyrazolic nitrogen atoms fall in the range 2.104(1)–2.128(2) Å, respectively. The N–N bond distances are in the range of 1.360(2)–1.364(2) Å. The bond angles on the equatorial plane range between 86.24(7)–94.22(7)°. The pyrazolic nitrogen atoms at axial positions are located at a distance of 2.117(2) and 2.106(2) Å and form angles with the metal centre that fall in the range of 78.10(7)–98.53(7)°, respectively. In a similar fashion, compound 3 crystalized in the monoclinic *P*2_1_/*c* space group. The unit cell contains four molecular species and two co-crystallized perchlorate counterions. The complex adopts an isostructural motif to the one observed in the 2 (Fig. 2). The Co(ii) centre is in the second oxidation state and occupy the centre of a distorted octahedral coordination sphere, which is completed by four nitrogen pyrazolic atoms provided by the two branches of the organic ligand one water and a methanol solvent molecule, respectively. The Co–O bond lengths observed in the water and methanol terminal ligands are found to be 2.120(2) and 2.108(2) Å while the distances between the Co(ii) metal centre and the pyrazolic nitrogen atoms fall in the range 2.123(2)–2.158(2) Å, respectively. All the Co–X bonds are found to be slightly elongated in comparison to complex 2. The pyrazolic N–N bond distances are in the range of 1.356(2)–1.359(3) Å. The bond angles on the equatorial plane range between 85.54(8)–94.94(9)°. The pyrazolic nitrogen atoms at axial positions are located at a distance of 2.155(2) and 2.158(2) Å, and form angles with the metal centre that fall in the range of 76.96(8)–99.80(8)°, respectively.

**Fig. 3:**
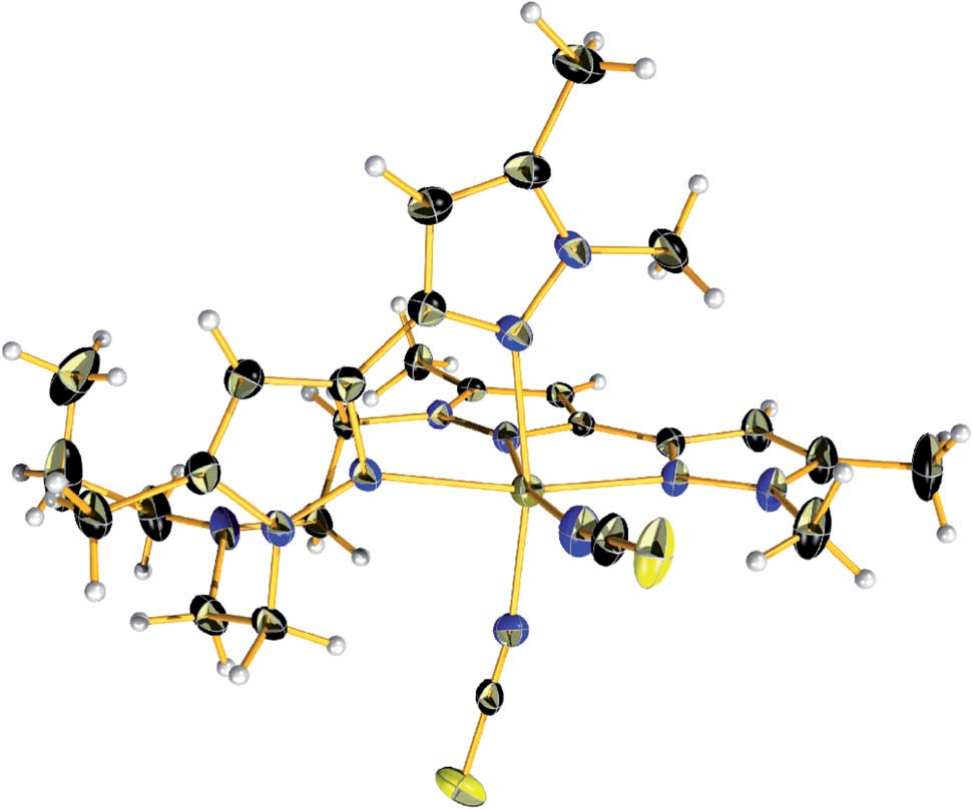
View of the molecular structure of [FeL(NCS)_2_] (4) at 150 K. Colour code: C, black; N, nitrogen; yellow, sulfur; light grey, hydrogen; olive green, iron.

Complex 4 crystalized in the *P*2_1_/*c* space group too but the unit cell contains four molecular species. The Fe(ii) centre is in the second oxidation state and occupy the centre of a distorted octahedral coordination sphere which is completed by four nitrogen pyrazolic atoms provided by the two branches of L and two SCN^−^ anions which are responsible for balancing the overall charge of the complex (Fig. 3). The bond lengths between the metal centre and the pyrazolic nitrogen donors (Fe–N_py_) fall within the range of 2.226(2) and 2.304(2) Å while the distances between the Fe(ii) metal centre and the N_SCN_ nitrogen atoms are found to be 2.085(2) – 2.099(2) Å, respectively. Compound 4 exhibit longer M–N_L_ distances compared to other reported complexes of this substance class, thus indicating a high-spin (HS) state for the Fe(ii) ions. Additionally, the bond angles on the equatorial plane range between 87.35(8)–96.7(1)°. The pyrazolic nitrogen atoms at axial positions are located at a distance of 2.274(2) and 2.304(2) Å and form angles with the metal centre that fall in the range of 73.87(7)–98.96(8)°, respectively. The local distortion parameter around the metal ion is *Σ*^HS^ = 73.42°.^44^ This structural parameter, defined as the sum of the deviations from 90° of the 12 *cis*-N–M–N angles, is useful to accurately evaluate the octahedral distortion of the MN_6_ coordination sphere.^45^

### 3.4. Spin state and magnetic investigation

**Fig. 4:**
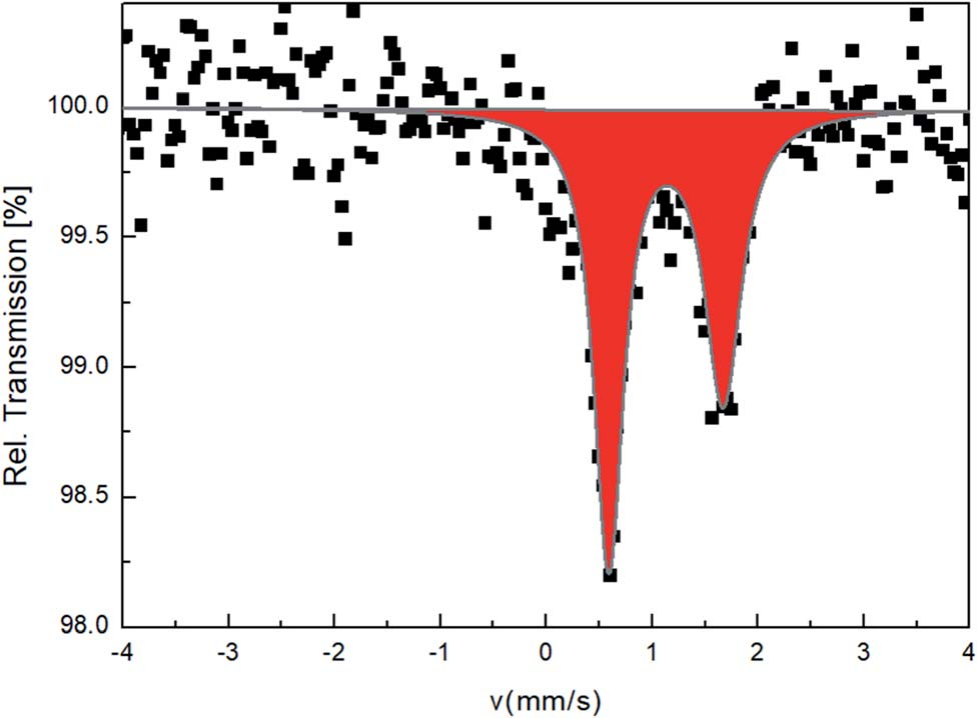
^57^Fe Mössbauer spectrum of 4 at 293 K recorded in transmission geometry.

A^57^Fe Mössbauer spectrum of 4 was recorded in transmission geometry at room temperature to probe the oxidation and spin state. The spectrum reveals one iron site with isomer shift *δ* = 1.13 mm s^−1^ and quadrupole splitting Δ*E*_Q_ = 1.08 mm s^−1^, characteristic of HS Fe(ii) ions (Fig. 4). This result is consistent with the one obtained by single crystal X-ray diffraction which revealed a single Fe(ii) site in the HS state too at 150 K. Such Δ*E*_Q_ is smaller compared to iron(ii) metallic salts, *e.g.* [Fe(H_2_O)_6_]SO_4_·7H_2_O which present a Δ*E*_Q_ = 3.2 mm s^−1^.^46^ Actually Δ*E*_Q_ is the result of two contributions to the electric field gradient originating from valence electrons (valence contribution) and to the local environment (lattice distortion), which is opposite in sign to the valence contribution.^47,48^ In the present case, the lattice contribution to the EFG is rather high, due to a distorted octahedron, which thereby decreases the quadrupole splitting value.

**Fig. 5:**
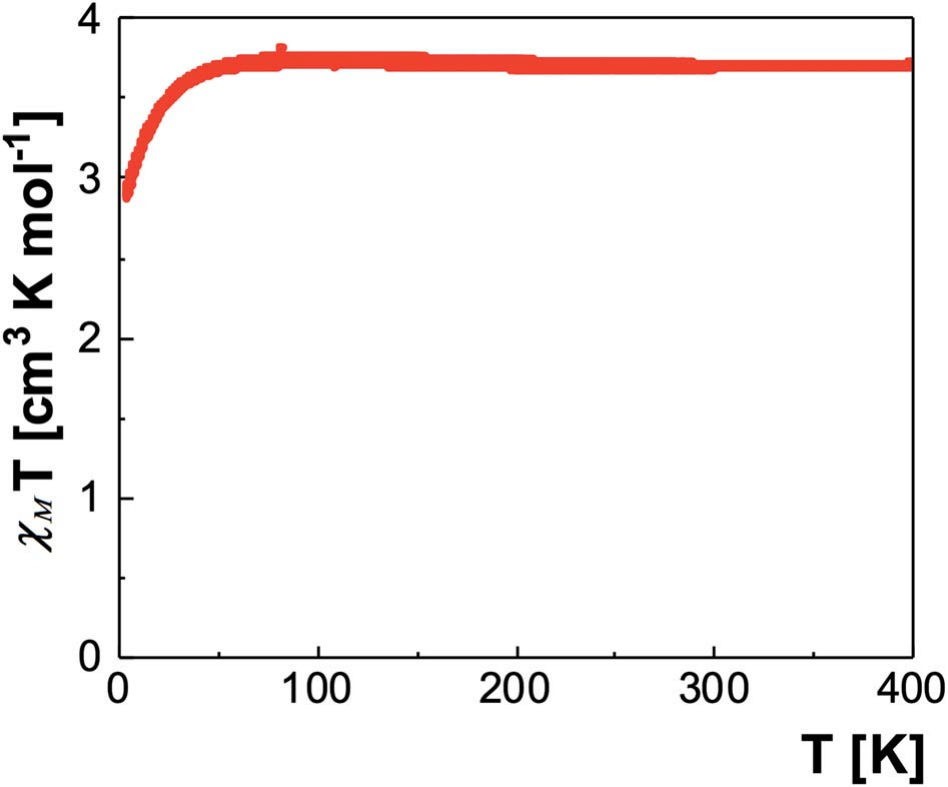
*χ*_M_ *T vs. T* for 4 over the temperature range 400–4.2 K. The measurement was repeated for three runs over the same temperature interval.

Given these spin state characteristics, a SQUID analysis was undertaken to determine whether complex 4 could switch its spin states below 150 K. At room temperature, *χ*_M_*T* = 3.71 cm^3^ K mol^−1^, which fits with Fe(ii) ions in the HS state, as well as with the Mössbauer result (Fig. 5). Upon cooling, the *χ*_M_*T* product remains constant even below 150 K, confirming a full HS state. Below 50 K, a decrease of *χ*_M_*T* is observed in agreement with the zero-field splitting of HS Fe(ii) ions.^49^ Thus complex 4 remains paramagnetic and do not display a thermally induced spin state crossover behaviour.

### 3.5. Antibacterial activities

**Fig. 6:**
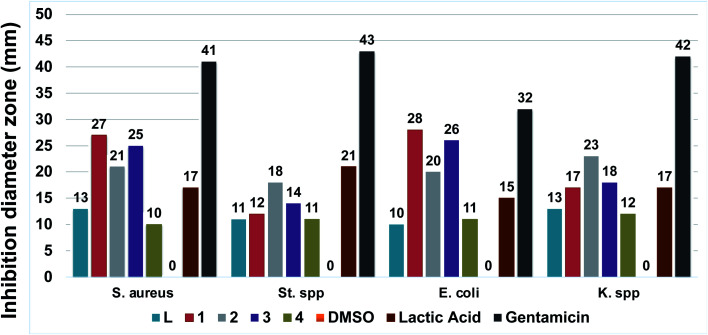
Antibacterial activities recap plot of L and the coordination complexes 1–4. Experiments were performed in triplicate.

The Ligand L and the coordination complexes 1–4 were tested against two Gram-positive bacteria (*Staphylococcus aureus* and *Streptococcus* spp.) and two Gram-negative bacteria (*Escherichia coli* and *Klebsiella* spp). The results are gathered in Table 1 and Fig. 6.

**Table 1:** Antibacterial activities of L and the complexes 1–4 against four strains (inhibition zones in mm)^a^

Compound	Bacteria			
	*S. aureus* ^[p]^	*St*. spp.^[p]^	*E. coli* ^[n]^	*K*. spp.^[n]^
L	13	11	10	13
1	27	12	28	17
2	21	18	20	23
3	25	14	26	18
4	10	11	11	12
DMSO	0	0	0	0
Lactic acid	17	21	15	17
Gentamicin	41	43	32	42

a ^[p]^: Gram-positive; ^[n]^: Gram-negative.

All compounds showed moderate to decent antibacterial activities, compared to literature reports. Complexes 1, 2 and 3 demonstrate noticeable improvements in term of inhibition compared to the ligand L and complex 4. In particular, complexes 1 and 3 reveal significant enhancement towards both Gram-positive *S. aureus* and Gram-negative *E. coli*. This is much higher compared to [Co(dmphen)X_2_] (dmphen = 2,9-dimethyl-1,10-phenanthroline) which was found to exhibit 17 and 18 mm for *E. coli* and.

*S. aureus* in the case of X = Br and 18 mm for both *E. coli* and *S. aureus* for X = NCS.^50^ The inhibition zone is much higher than the positive control lactic acid too. Both of these complexes exhibit modest inhibition increase against *Klebsiella* spp bacteria, to match the same score as lactic acid. On the other hand, complex 2 affords an inhibition jump up in all tested bacteria, especially the Gram-negative *Klebsiella* spp. which was again greater than lactic acid.

In conclusion, the best recorded results for *S. aureus* were claimed for 1 and 3, which show 27 and 25 mm inhibition zones, respectively. The highest attitude against Streptococcus spp was found for 2 with a decent 18 inhibition zone. Meanwhile 1 and 3 earned once again the steepest inhibition zone for *E. coli* being 28 and 26 mm, respectively. Finally, regarding *Klebsiella* spp., the top fruition was reached by 2 with 23 mm. These results were compared to several reported ligand and complexes with potential antibacterial behaviors and proved to be better to competitive.^51–53^

### 3.6. Antifungal activities

**Fig. 7:**
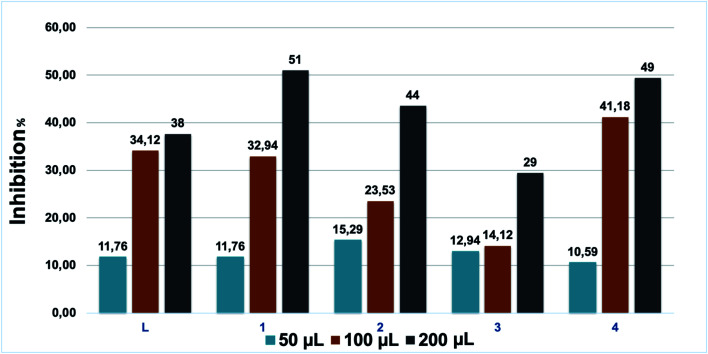
Antifungal activities of L and the coordination complexes 1–4. Experiments were performed in triplicate.

The antifugal activities of our compounds were also investigated *vs. Fusarium oxysporum* f.sp. *albedinis* (Fig. 7), which is a real threat in modern forestry, in particular to date palm (*Phoenix dactylifera*).^54^

According to Table 2, all tested samples present considerable anti-Fusarium activities, but complexes 1 and 4 can be distinguished. These results are quite expected due to the fact that the use of coordination complexes is assisted by the improvement of the overall therapeutic activities.^55^ The complexation of L with copper and iron metal ions leads to an increase in the lipophilicity and a simultaneous expansion of the hydrocarbon portion. In addition, chelation lowers the polarity of the metal ion due to the partial sharing of its positive charge with the donor groups in the coordination complexes. This process raises the lipophilicity of the metal complexes and thus facilitates their permeability through the lipid bilayer of membranes.^56^ To confirm this effect, these complexes were tested three times to determine the necessary volume to reach a 50% inhibition: 32 and 83.5 μmol L^−1^ were needed for 1 and 4, respectively. These results are superior to literature results for many pyrazole derivatives.^57–59^

**Table 2:** Antifungal activities of L and the complexes 1–4

Compound	Sample volumes (μL)	Concentration (μmol L^−1^)	*D*_*x*_ (cm)	Inhibition (%)
L	50	28	7.5	11.76
	100	58.2	5.6	34.12
	200	114.3	5.3	38
1	50	8.5	7.5	11.76
	100	17.7	5.7	32.94
	200	34.8	4.2	51
2	50	16.9	7.2	15.29
	100	35.1	6.5	23.53
	200	68.8	4.8	44
3	50	16.8	7.4	12.94
	100	3.5	7.3	14.12
	200	68.7	6	29
4	50	20.5	7.6	10.59
	100	42.5	5	41.18
	200	83.5	4.3	49

## 4. Conclusions

In summary, we have brought a new class of molecules based on pyrazole rings. The synthesis of such molecules remains challenging in most cases giving the limited literature available. Yet, we have established a simple method that awarded us the ligand L with high nitrogen donors and high flexibility. Four coordination complexes were synthesised with different topologies, which was established by single crystal X-ray diffraction. The antibacterial activities performed on both Gram-positive as wells as Gram-negative bacteria, revealed competitive properties compared to existing literature, in particular for 1 and 3. In addition, remarkable antifungal behaviours towards *Fusarium oxysporum* f.sp. *albedinis* fungi were obtained for 1 and 4.

## Author contributions

YD performed synthesis, characterization and wrote the paper with YG and HNM. SR and YG both designed and managed the project. AT and AO run some experiments. HNM undertook all X-ray studies. RB, SO and SM performed antibacterial and antifungal experiments. AR recorded SQUID measurements.

## Conflicts of interest

There are no conflicts to declare.

## Supplementary Material

RA-012-D2RA03414J-s001

RA-012-D2RA03414J-s002

## References

[cit1] Nakahara Y., Toda T., Matsunami A., Kayaki Y., Kuwata S. (2018). Chem.–Asian J..

[cit2] Tsygankova V., Ya A., Shtompel O., Romaniuk O., Yaikova M., Hurenko A., Solomyanny R., Abdurakhmanova E., Klyuchko S., Holovchenko O. (2017). J. Med. Biotechnol. Genetics S..

[cit3] Sayed G. H., Azab M. E., Anwer K. E., Raouf M. A., Negm N. A. (2018). J. Mol. Liq..

[cit4] Ansari A., Ali A., Asif M. (2017). New J. Chem..

[cit5] Karrouchi K., Radi S., Ramli Y., Taoufik J., Mabkhot Y. N., Al-Aizari F. A., Ansar M. h. (2018). Molecules.

[cit6] Chougala B. M., Samundeeswari S., Holiyachi M., Shastri L. A., Dodamani S., Jalalpure S., Dixit S. R., Joshi S. D., Sunagar V. A. (2017). Eur. J. Med. Chem..

[cit7] Nossier E. S., Fahmy H. H., Khalifa N. M., El-Eraky W. I., Baset M. A. (2017). Molecules.

[cit8] Oulmidi A., Radi S., Idir A., Zyad A., Kabach I., Nhiri M., Robeyns K., Rotaru A., Garcia Y. (2021). RSC Adv..

[cit9] Usami Y., Tsujiuchi Y., Machiya Y., Chiba A., Ikawa T., Yoneyama H., Harusawa S. (2020). Heterocycles.

[cit10] Ali I. O., Salama T. M., Bakr M. F., El-Henawy A. A., Lateef M. A., Guma H. A. (2018). Res. Chem. Intermed..

[cit11] Oulmidi A., Radi S., Miras H. N., Adarsh N. N., Garcia Y. (2020). Sustainability.

[cit12] Adarsh N., Kumar D. K., Dastidar P. (2009). CrystEngComm.

[cit13] Bikas R., Kuncser V., Sanchiz J., Schinteie G., Siczek M., Hosseini-Monfared H., Lis T. (2018). Polyhedron.

[cit14] Zhang Z., Zaworotko M. J. (2014). Chem. Soc. Rev..

[cit15] Kumar V., El-Massaoudi M., Radi S., Van Hecke K., Rotaru A., Garcia Y. (2020). New J. Chem..

[cit16] Radi S., El-Massaoudi M., Benaissa H., Adarsh N., Ferbinteanu M., Devlin E., Sanakis Y., Garcia Y. (2017). New J. Chem..

[cit17] Karrouchi K., Radi S., Ramli Y., Taoufik J., Mabkhot Y. N., Al-Aizari F. A., Ansar M. h. (2018). Molecules.

[cit18] Tigreros A., Portilla J. (2020). RSC Adv..

[cit19] Cetin A., Korkmaz A., Bildirici I. (2018). Colloid Polym..

[cit20] Chkirate K., Fettach S., Karrouchi K., Sebbar N. K., Essassi E. M., Mague J. T., Radi S., Faouzi M. E. A., Adarsh N., Garcia Y. (2019). J. Inorg. Biochem..

[cit21] Kulmaczewski R., Bamiduro F., Shahid N., Cespedes O., Halcrow M. A. (2021). Chem.–Eur. J..

[cit22] Oulmidi A., Rotaru A., Radi S., Garcia Y. (2021). Hyperfine Interact..

[cit23] Lin H.-J., Lutz S., O'Kane C., Zeller M., Chen C.-H., Al Assil T., Lee W.-T. (2018). Dalton Trans..

[cit24] Mandal S., Mondal M., Biswas J. K., Cordes D. B., Slawin A. M., Butcher R. J., Saha M., Saha N. C. (2018). J. Mol. Struct..

[cit25] Czarnomysy R., Surażyński A., Muszynska A., Gornowicz A., Bielawska A., Bielawski K. (2018). J. Enzyme Inhib. Med. Chem..

[cit26] Paitandi R. P., Mukhopadhyay S., Singh R. S., Sharma V., Mobin S. M., Pandey D. S. (2017). Inorg. Chem..

[cit27] Chkirate K., Karrouchi K., Dege N., Sebbar N. K., Ejjoummany A., Radi S., Adarsh N., Talbaoui A., Ferbinteanu M., Essassi E. M. (2020). New J. Chem..

[cit28] Jodeh S., Hanbali G., Tighadouini S., Radi S., Hamed O., Jodeh D. (2019). BMC Chem..

[cit29] El-Massaoudi M., Radi S., Lamsayah M., Tighadouini S., Séraphin K. K., Kouassi L. K., Garcia Y. (2021). J. Cleaner Prod..

[cit30] Radi S., El Massaoudi M., Bacquet M., Degoutin S., Adarsh N., Robeyns K., Garcia Y. (2017). Inorg. Chem. Front..

[cit31] Lagarec K. and Rancourt D., Recoil - Mössbauer Spectral Analysis Software for Windows, version 1.0, University of Ottawa, Ottawa, ON, 1998

[cit32] Sheldrick G. M. (1990). Acta Crystallogr., Sect. A: Found. Crystallogr..

[cit33] Sheldrick G. M. (2008). Acta Crystallogr., Sect. A: Found. Crystallogr..

[cit34] Farrugia L. J. (1999). J. Appl. Crystallogr..

[cit35] Clark R., Reid J. (1995). Acta Crystallogr., Sect. A: Found. Crystallogr..

[cit36] Attayibat A., Radi S., Lekchiri Y., Ramdani A., Hacht B., Morcellet M., Bacquet M., Willai S. (2006). J. Chem. Res..

[cit37] Hurst J. K. (2005). Coord. Chem. Rev..

[cit38] Chatterjee D., Mitra A., Shepherd R. E. (2004). Inorg. Chim. Acta.

[cit39] Chafi A., Benabbes R., Bouakka M., Hakkou A., Kouddane N., Berrichi A. (2015). J. Mater. Environ. Sci..

[cit40] Neri F., Mari M., Brigati S. (2006). Plant Pathol..

[cit41] Hmouni A., Hajlaoui M., Mlaiki A. (1996). EPPO Bull..

[cit42] Onishi N., Kanega R., Fujita E., Himeda Y. (2019). Adv. Synth. Catal..

[cit43] Jaćimović Ž. K., Giester G., Kosović M., Bogdanović G. A., Novaković S. B., Leovac V. M., Latinović N., Hollo B. B., Szecsenyi K. M. (2017). J. Therm. Anal. Calorim..

[cit44] Ketkaew R., Tantirungrotechai Y., Harding P., Chastanet G., Guionneau P., Marchivie M., Harding D. J. (2021). Dalton Trans..

[cit45] Garcia Y., Bravic G., Chasseau D., Gieck C., Tremel W., Gütlich P. (2005). Inorg. Chem..

[cit46] DeBenedetti S., Lang G., Ingalls R. (1961). Phys. Rev. Lett..

[cit47] Gütlich P. and Garcia Y., in Mössbauer spectroscopy – Tutorial Book, ed. Y. Yoshida and G. Langouche. Springer, 2013, ch. 2, pp. 23–89

[cit48] Gütlich P., Garcia Y. (2010). J. Phys.: Conf. Ser..

[cit49] Garcia Y., Grunert C. M., Reiman S., Van Campenhoudt O., Gütlich P. (2006). Eur. J. Inorg. Chem..

[cit50] Al-Noaimi M., Awwadi F. F., Haddad S. F., Talib W. H., Jodeh S., Radi S., Hadda T. B., Abdoh M., Naveen S., Lokanath N. (2015). J. Mol. Struct..

[cit51] Mabkhot Y. N., Al-Majid A. M., Barakat A., Al-Showiman S. S., Al-Har M. S., Radi S., Naseer M. M., Hadda T. B. (2014). Int. J. Mol. Sci..

[cit52] El Shehry M. F., Ghorab M. M., Abbas S. Y., Fayed E. A., Shedid S. A., Ammar Y. A. (2018). Eur. J. Med. Chem..

[cit53] Tighadouini S., Radi S., Benabbes R., Youssoufi M. H., Shityakov S., El Massaoudi M., Garcia Y. (2020). ACS Omega.

[cit54] Komeil D. A., Abdalla M. Y., El-Bebany A. F., Basyony A. B. A. (2021). Asian J. Plant Sci..

[cit55] Ali I., Wani W. A., Khan A., Haque A., Ahmad A., Saleem K., Manzoor N. (2012). Microb. Pathog..

[cit56] Chohan Z. H., Pervez H., Khan K. M., Rauf A., Maharvi G. M., Supuran C. T. (2004). J. Enzyme Inhib. Med. Chem..

[cit57] Tighadouini S., Radi S., Abrigach F., Benabbes R., Eddike D., Tillard M. (2019). J. Chem. Inf. Model..

[cit58] Kaddouri Y., Abrigach F., Ouahhoud S., Benabbes R., El Kodadi M., Alsalme A., Al-Zaqri N., Warad I., Touzani R. (2020). Front. Chem..

[cit59] Mondal G., Jana H., Acharjya M., Santra A., Bera P., Jana A., Panja A., Bera P. (2017). Med. Chem. Res..

