# Supplementary material for: Novel family of bis-pyrazole coordination complexes as potent antibacterial and antifungal agents
Source: RSC Adv. 2022 Jun 15;12(28):17755–64. doi: 10.1039/d2ra03414j (PMC9198996; doi:10.1039/d2ra03414j)
Supplement: RA-012-D2RA03414J-s001 [file RA-012-D2RA03414J-s001.pdf]

Electronic Supplementary Material (ESI) for RSC Advances.
This journal is © The Royal Society of Chemistry 2022

Electronic Supplementary Information (ESI)

Novel Family of Bi-pyrazole Tripodal Based Coordination Complexes as Potent Antibacterial and Antifungal agents

Youssef Draoui [1], Smaail Radi [*,1], Amine Tanan [1], Afaf Oulmidi [1,2] Haralampos N. Miras [3] Redouane Benabbes [4], Sabir Ouahhoudo [4], Samira Mamri [4] Aurelian Rotaru [5], Yann Garcia[*,2]

[1] *LCAE, Department of Chemistry, Faculty of Science, University Mohamed I , P.O. Box 524, Oujda 60 000, Morocco.*

[2] *Institute of Condensed Matter and Nanosciences, Molecular Chemistry, Materials and Catalysis (IMCN/MOST), Université catholique de Louvain, Belgium*

[3] *School of Chemistry, Joseph Black Building, University of Glasgow, Glasgow G12 8QQ, UK.*

[4] *Laboratory of Biochemistry and Biotechnology, Department of Biology, Faculty of Science, University Mohamed I , P.O. Box 524, Oujda 60 000, Morocco.*

[5] *Department of Electrical Engineering and Computer Science & Research Center MANSiD, "Stefan cel Mare" University, University Street, No. 13, Suceava 720229, Romania*

Fax: +32-10472330 E-mail: yann.garcia@uclouvain.be ; s.radi@ump.ac.ma

Contents:

Scheme S1. Synthetic routes of **L**. *(i) MeI/t-BuOK/*Et_2O*. (ii) Acetone/Na/Toluene. (iii)* N_2H_4*,*H_2O*/EtOH. (iv) Br-*CH_2*-*CO_2Et*/t-BuOK/ THF. (v)* $LiAlH_4$*/THF. (vi)* $TsCl_2$*/*CH_2Cl_2*. (vii) Propylamine/*K_2CO_3*/*CH_3CN*.*

Table S1. Crystallographic and refinement data for **1-4**.

Table S2. Bond lengths [Å] and angles [°] for **1.**

Table S3. Bond lengths [Å] and angles [°] for **2.**

Table S4. Bond lengths [Å] and angles [°] for **3.**

Table S5. Bond lengths [Å] and angles [°] for **4.**

Fig. S1 FT-IR spectroscopy comparison plot of **L** with **1** - **4**.

Fig. S2 Diffuse reflectance spectroscopy comparison plot of **L** with **1** - **4**.

NMR of **L**

HRMS of **L**, **1**, **2**, **3** and **4**.

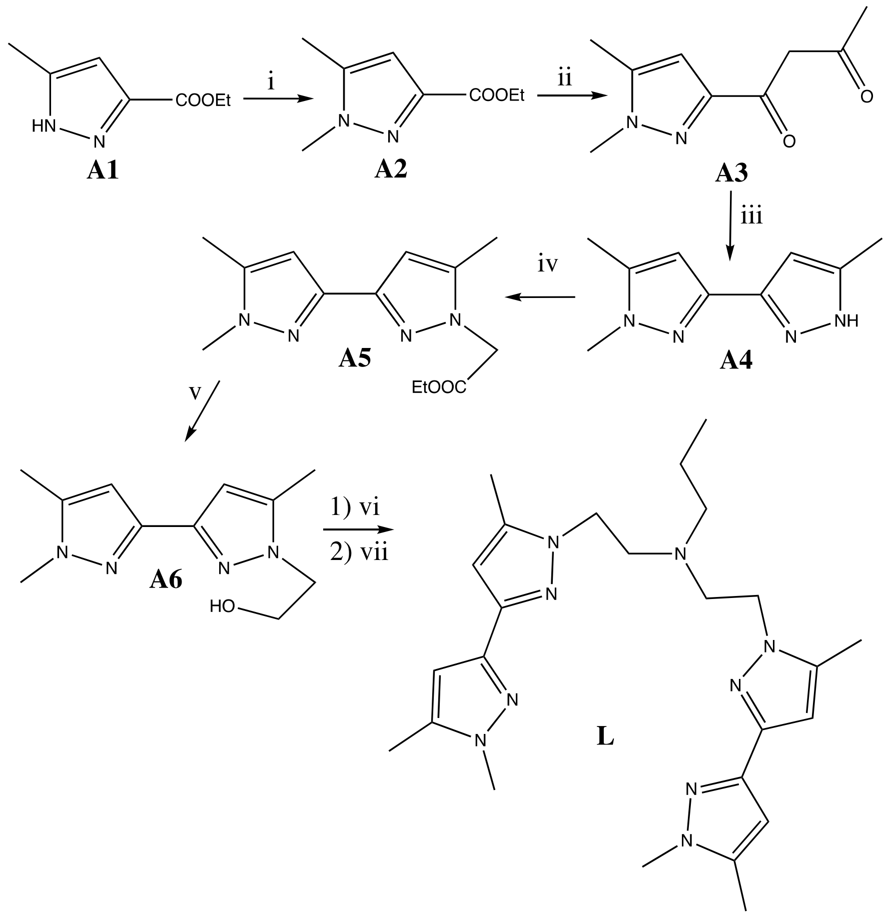

Scheme S1. Synthetic routes of **L**. *(i) MeI/t-BuOK/*Et_2O*. (ii) Acetone/Na/Toluene. (iii)* N_2H_4*,*H_2O*/EtOH. (iv) Br-*CH_2*-*CO_2Et*/t-BuOK/THF. (v)* $LiAlH_4$*/THF. (vi)* $TsCl_2$*/*CH_2Cl_2*. (vii) Propylamine/*K_2CO_3*/*CH_3CN*.*

Table S1. Crystallographic and refinement data for **1-4**.

	**1**	**2**	**3**	**4**
Formula	$C_{51}H_{74}Cl_8Cu_4N_{18}O_3$	$C_{26}H_{42}Cl_2N_9NiO_{10}$	$C_{26}H_{43}Cl_2CoN_9O_{10}$	$C_{27}H_{37}FeN_{11}S_2$
M_r	1525.08	770.29	771.52	635.64
T [K]	150(2)	150(2)	150(2)	150(2)
λ [Å]	0.71073	0.71073	0.71073	0.71073
Crystal system	Triclinic	Monoclinic	Monoclinic	Monoclinic
Space group	*P*-1	*P*2$_1$/*c*	*P*2$_1$/*c*	*P*2$_1$/*c*
a [Å]	8.717(1)	10.909(2)	10.900(3)	11.817(4)
b [Å]	13.818(2)	21.821(5)	21.782(5)	21.125(7)
c [Å]	14.386(3)	14.531(3)	14.480(3)	14.003(5)
α [°]	81.029(2)	90	90	90
β [°]	73.073(2)	98.616(3)	98.704(3)	111.244(5)
γ [°]	89.881(2)	90	90	90
V [Å^3]	1635.8(6)	3420.2(1)	3398.1(1)	3258(2)
Z	1	4	4	4
ρ_c [g cm^{-3}]	1.548	1.496	1.508	1.296
μ [mm^{-1}]	1.664	0.790	0.729	0.627
F(000)	782	1612	1612	1336
Independent reflns	6218	6342	5598	4752
Abs. correction	Empirical	Empirical	Empirical	Empirical
Refinement method	Full-matrix least-squares on F^2			
GoF on F^2	1.047	1.022	1.042	1.034
Final R indices [I > 2 σ(I)]	R_1 = 0.0256<br>wR_2 = 0.066	R_1 = 0.0449<br>wR_2 = 0.1191	R_1 = 0.0495<br>wR_2 = 0.1208	R_1 = 0.0448<br>wR_2 = 0.1033
R indices (all data)	R_1 = 0.0290<br>wR_2 = 0.069	R_1 = 0.0524<br>wR_2 = 0.1261	R_1 = 0.0671<br>wR_2 = 0.1406	R1 = 0.0768<br>wR_2 = 0.1171

Table S2. Bond lengths [Å] and angles [°] for **1.**

C(1)-N(6)	1.454(3)
C(1)-H(1A)	0.9800
C(1)-H(1B)	0.9800
C(1)-H(1C)	0.9800
C(2)-C(4)	1.494(3)
C(2)-H(2A)	0.9800
C(2)-H(2B)	0.9800
C(2)-H(2C)	0.9800
N(6)-N(5)	1.356(2)
N(6)-C(4)	1.358(3)
C(4)-C(5)	1.381(3)
C(5)-C(3)	1.395(3)
C(5)-H(5)	0.9500
C(3)-N(5)	1.342(2)
C(3)-C(7)	1.453(3)
C(7)-N(7)	1.340(2)
C(7)-C(8)	1.398(3)
C(8)-C(9)	1.387(3)
C(8)-H(8)	0.9500
C(9)-N(8)	1.360(2)
C(9)-C(10)	1.494(3)
C(10)-H(10A)	0.9800
C(10)-H(10B)	0.9800
C(10)-H(10C)	0.9800
N(8)-N(7)	1.348(2)
N(8)-C(12)	1.460(2)
C(12)-C(13)	1.526(3)
C(12)-H(12A)	0.9900
C(12)-H(12B)	0.9900
C(13)-N(9)	1.508(2)

C(13)-H(13A)	0.9900
C(13)-H(13B)	0.9900
N(9)-C(15)	1.500(2)
N(9)-C(18)	1.500(2)
N(9)-Cu(2)	2.1200(16)
C(15)-C(16)	1.527(3)
C(15)-H(15A)	0.9900
C(15)-H(15B)	0.9900
C(16)-C(17)	1.522(3)
C(16)-H(16A)	0.9900
C(16)-H(16B)	0.9900
C(17)-H(17A)	0.9800
C(17)-H(17B)	0.9800
C(17)-H(17C)	0.9800
C(18)-C(19)	1.530(3)
C(18)-H(18A)	0.9900
C(18)-H(18B)	0.9900
C(19)-N(4)	1.463(2)
C(19)-H(19A)	0.9900
C(19)-H(19B)	0.9900
N(4)-C(21)	1.354(2)
N(4)-N(3)	1.363(2)
C(21)-C(23)	1.388(3)
C(21)-C(22)	1.496(3)
C(22)-H(22A)	0.9800
C(22)-H(22B)	0.9800
C(22)-H(22C)	0.9800
C(23)-C(24)	1.396(3)
C(23)-H(23)	0.9500
C(24)-N(3)	1.342(2)
C(24)-C(25)	1.458(3)
C(25)-N(2)	1.344(2)
C(25)-C(26)	1.392(3)

C(26)-C(27)	1.382(3)
C(26)-H(26)	0.9500
C(27)-N(1)	1.355(3)
C(27)-C(28)	1.497(3)
C(28)-H(28A)	0.9800
C(28)-H(28B)	0.9800
C(28)-H(28C)	0.9800
N(1)-N(2)	1.361(2)
N(1)-C(30)	1.457(3)
C(30)-H(30A)	0.9800
C(30)-H(30B)	0.9800
C(30)-H(30C)	0.9800
N(2)-Cu(1)	2.0132(16)
N(3)-Cu(1)	2.0202(15)
N(7)-Cu(2)	2.0393(16)
N(5)-Cu(2)	2.0522(16)
Cl(1)-Cu(1)	2.2072(6)
Cl(2)-Cu(1)	2.2062(6)
Cl(3)-Cu(2)	2.3419(6)
Cl(4)-Cu(2)	2.3763(6)
C(31)-O(1B)	1.087(10)

N(6)-C(1)-H(1A)	109.5
N(6)-C(1)-H(1B)	109.5
H(1A)-C(1)-H(1B)	109.5
N(6)-C(1)-H(1C)	109.5
H(1A)-C(1)-H(1C)	109.5
H(1B)-C(1)-H(1C)	109.5
C(4)-C(2)-H(2A)	109.5
C(4)-C(2)-H(2B)	109.5
H(2A)-C(2)-H(2B)	109.5
C(4)-C(2)-H(2C)	109.5
H(2A)-C(2)-H(2C)	109.5

H(2B)-C(2)-H(2C)	109.5
N(5)-N(6)-C(4)	110.88(16)
N(5)-N(6)-C(1)	120.72(16)
C(4)-N(6)-C(1)	128.39(17)
N(6)-C(4)-C(5)	107.34(17)
N(6)-C(4)-C(2)	122.11(19)
C(5)-C(4)-C(2)	130.55(19)
C(4)-C(5)-C(3)	105.21(17)
C(4)-C(5)-H(5)	127.4
C(3)-C(5)-H(5)	127.4
N(5)-C(3)-C(5)	110.76(17)
N(5)-C(3)-C(7)	114.18(16)
C(5)-C(3)-C(7)	134.97(18)
N(7)-C(7)-C(8)	110.29(17)
N(7)-C(7)-C(3)	113.67(16)
C(8)-C(7)-C(3)	135.96(17)
C(9)-C(8)-C(7)	105.52(16)
C(9)-C(8)-H(8)	127.2
C(7)-C(8)-H(8)	127.2
N(8)-C(9)-C(8)	106.65(16)
N(8)-C(9)-C(10)	121.81(18)
C(8)-C(9)-C(10)	131.51(17)
C(9)-C(10)-H(10A)	109.5
C(9)-C(10)-H(10B)	109.5
H(10A)-C(10)-H(10B)	109.5
C(9)-C(10)-H(10C)	109.5
H(10A)-C(10)-H(10C)	109.5
H(10B)-C(10)-H(10C)	109.5
N(7)-N(8)-C(9)	111.22(16)
N(7)-N(8)-C(12)	119.88(15)
C(9)-N(8)-C(12)	128.87(16)
N(8)-C(12)-C(13)	112.93(15)
N(8)-C(12)-H(12A)	109.0

C(13)-C(12)-H(12A)	109.0
N(8)-C(12)-H(12B)	109.0
C(13)-C(12)-H(12B)	109.0
H(12A)-C(12)-H(12B)	107.8
N(9)-C(13)-C(12)	117.43(16)
N(9)-C(13)-H(13A)	107.9
C(12)-C(13)-H(13A)	107.9
N(9)-C(13)-H(13B)	107.9
C(12)-C(13)-H(13B)	107.9
H(13A)-C(13)-H(13B)	107.2
C(15)-N(9)-C(18)	111.58(14)
C(15)-N(9)-C(13)	112.12(14)
C(18)-N(9)-C(13)	104.54(14)
C(15)-N(9)-Cu(2)	109.09(11)
C(18)-N(9)-Cu(2)	113.53(11)
C(13)-N(9)-Cu(2)	105.82(11)
N(9)-C(15)-C(16)	116.44(15)
N(9)-C(15)-H(15A)	108.2
C(16)-C(15)-H(15A)	108.2
N(9)-C(15)-H(15B)	108.2
C(16)-C(15)-H(15B)	108.2
H(15A)-C(15)-H(15B)	107.3
C(17)-C(16)-C(15)	110.70(16)
C(17)-C(16)-H(16A)	109.5
C(15)-C(16)-H(16A)	109.5
C(17)-C(16)-H(16B)	109.5
C(15)-C(16)-H(16B)	109.5
H(16A)-C(16)-H(16B)	108.1
C(16)-C(17)-H(17A)	109.5
C(16)-C(17)-H(17B)	109.5
H(17A)-C(17)-H(17B)	109.5
C(16)-C(17)-H(17C)	109.5
H(17A)-C(17)-H(17C)	109.5

H(17B)-C(17)-H(17C)	109.5
N(9)-C(18)-C(19)	114.80(15)
N(9)-C(18)-H(18A)	108.6
C(19)-C(18)-H(18A)	108.6
N(9)-C(18)-H(18B)	108.6
C(19)-C(18)-H(18B)	108.6
H(18A)-C(18)-H(18B)	107.5
N(4)-C(19)-C(18)	108.75(15)
N(4)-C(19)-H(19A)	109.9
C(18)-C(19)-H(19A)	109.9
N(4)-C(19)-H(19B)	109.9
C(18)-C(19)-H(19B)	109.9
H(19A)-C(19)-H(19B)	108.3
C(21)-N(4)-N(3)	110.87(15)
C(21)-N(4)-C(19)	128.54(16)
N(3)-N(4)-C(19)	120.40(15)
N(4)-C(21)-C(23)	107.05(16)
N(4)-C(21)-C(22)	122.79(18)
C(23)-C(21)-C(22)	130.16(18)
C(21)-C(22)-H(22A)	109.5
C(21)-C(22)-H(22B)	109.5
H(22A)-C(22)-H(22B)	109.5
C(21)-C(22)-H(22C)	109.5
H(22A)-C(22)-H(22C)	109.5
H(22B)-C(22)-H(22C)	109.5
C(21)-C(23)-C(24)	105.59(17)
C(21)-C(23)-H(23)	127.2
C(24)-C(23)-H(23)	127.2
N(3)-C(24)-C(23)	110.40(16)
N(3)-C(24)-C(25)	114.82(16)
C(23)-C(24)-C(25)	134.73(17)
N(2)-C(25)-C(26)	110.44(16)
N(2)-C(25)-C(24)	114.94(16)

C(26)-C(25)-C(24)	134.56(17)
C(27)-C(26)-C(25)	105.58(17)
C(27)-C(26)-H(26)	127.2
C(25)-C(26)-H(26)	127.2
N(1)-C(27)-C(26)	107.33(16)
N(1)-C(27)-C(28)	122.33(18)
C(26)-C(27)-C(28)	130.34(18)
C(27)-C(28)-H(28A)	109.5
C(27)-C(28)-H(28B)	109.5
H(28A)-C(28)-H(28B)	109.5
C(27)-C(28)-H(28C)	109.5
H(28A)-C(28)-H(28C)	109.5
H(28B)-C(28)-H(28C)	109.5
C(27)-N(1)-N(2)	110.68(15)
C(27)-N(1)-C(30)	128.28(16)
N(2)-N(1)-C(30)	121.03(16)
N(1)-C(30)-H(30A)	109.5
N(1)-C(30)-H(30B)	109.5
H(30A)-C(30)-H(30B)	109.5
N(1)-C(30)-H(30C)	109.5
H(30A)-C(30)-H(30C)	109.5
H(30B)-C(30)-H(30C)	109.5
C(25)-N(2)-N(1)	105.98(15)
C(25)-N(2)-Cu(1)	115.02(12)
N(1)-N(2)-Cu(1)	138.85(13)
C(24)-N(3)-N(4)	106.07(14)
C(24)-N(3)-Cu(1)	115.05(12)
N(4)-N(3)-Cu(1)	138.88(12)
C(7)-N(7)-N(8)	106.32(15)
C(7)-N(7)-Cu(2)	117.68(13)
N(8)-N(7)-Cu(2)	135.86(12)
C(3)-N(5)-N(6)	105.81(15)
C(3)-N(5)-Cu(2)	116.85(13)

N(6)-N(5)-Cu(2)	137.18(13)
N(2)-Cu(1)-N(3)	79.84(6)
N(2)-Cu(1)-Cl(2)	101.39(5)
N(3)-Cu(1)-Cl(2)	138.75(5)
N(2)-Cu(1)-Cl(1)	142.30(5)
N(3)-Cu(1)-Cl(1)	101.94(5)
Cl(2)-Cu(1)-Cl(1)	101.10(2)
N(7)-Cu(2)-N(5)	76.79(6)
N(7)-Cu(2)-N(9)	87.43(6)
N(5)-Cu(2)-N(9)	164.07(6)
N(7)-Cu(2)-Cl(3)	130.03(5)
N(5)-Cu(2)-Cl(3)	91.24(5)
N(9)-Cu(2)-Cl(3)	97.46(5)
N(7)-Cu(2)-Cl(4)	115.07(5)
N(5)-Cu(2)-Cl(4)	93.73(5)
N(9)-Cu(2)-Cl(4)	94.87(5)
Cl(3)-Cu(2)-Cl(4)	113.98(2)

Table S3. Bond lengths [Å] and angles [°] for **2.**

C(1)-N(1)	1.458(3)
C(1)-H(1A)	0.9800
C(1)-H(1B)	0.9800
C(1)-H(1C)	0.9800
C(2)-C(3)	1.499(4)
C(2)-H(2A)	0.9800
C(2)-H(2B)	0.9800
C(2)-H(2C)	0.9800
C(3)-N(1)	1.358(3)
C(3)-C(4)	1.382(4)
C(4)-C(5)	1.402(3)

C(4)-H(4)	0.9500
C(5)-N(2)	1.345(3)
C(5)-C(6)	1.455(3)
C(6)-N(3)	1.347(3)
C(6)-C(7)	1.400(3)
C(7)-C(8)	1.384(4)
C(7)-H(7)	0.9500
C(8)-N(4)	1.365(3)
C(8)-C(9)	1.493(3)
C(9)-H(9A)	0.9800
C(9)-H(9B)	0.9800
C(9)-H(9C)	0.9800
C(10)-N(4)	1.461(3)
C(10)-C(11)	1.532(3)
C(10)-H(10A)	0.9900
C(10)-H(10B)	0.9900
C(11)-N(5)	1.477(3)
C(11)-H(11A)	0.9900
C(11)-H(11B)	0.9900
C(12)-N(5)	1.496(3)
C(12)-C(13)	1.531(3)
C(12)-H(12A)	0.9900
C(12)-H(12B)	0.9900
C(13)-C(14)	1.521(4)
C(13)-H(13A)	0.9900
C(13)-H(13B)	0.9900
C(14)-H(14A)	0.9800
C(14)-H(14B)	0.9800
C(14)-H(14C)	0.9800
C(15)-N(5)	1.501(3)
C(15)-C(16)	1.531(3)
C(15)-H(15A)	0.9900
C(15)-H(15B)	0.9900

C(16)-N(6)	1.456(3)
C(16)-H(16A)	0.9900
C(16)-H(16B)	0.9900
C(17)-C(18)	1.495(3)
C(17)-H(17A)	0.9800
C(17)-H(17B)	0.9800
C(17)-H(17C)	0.9800
C(18)-N(6)	1.365(3)
C(18)-C(19)	1.382(4)
C(19)-C(20)	1.399(3)
C(19)-H(19)	0.9500
C(20)-N(7)	1.344(3)
C(20)-C(21)	1.455(3)
C(21)-N(8)	1.342(3)
C(21)-C(22)	1.401(3)
C(22)-C(23)	1.388(4)
C(22)-H(22)	0.9500
C(23)-N(9)	1.356(3)
C(23)-C(24)	1.499(3)
C(24)-H(24A)	0.9800
C(24)-H(24B)	0.9800
C(24)-H(24C)	0.9800
C(25)-N(9)	1.457(3)
C(25)-H(25A)	0.9800
C(25)-H(25B)	0.9800
C(25)-H(25C)	0.9800
C(26)-O(10)	1.439(3)
C(26)-H(26A)	0.9800
C(26)-H(26B)	0.9800
C(26)-H(26C)	0.9800
N(1)-N(2)	1.363(3)
N(2)-Ni(1)	2.1064(19)
N(3)-N(4)	1.361(3)

N(3)-Ni(1)	2.1044(19)
N(6)-N(7)	1.364(3)
N(7)-Ni(1)	2.1175(19)
N(8)-N(9)	1.366(3)
N(8)-Ni(1)	2.1282(19)
Ni(1)-O(10)	2.0920(16)
Ni(1)-O(9)	2.0995(18)
O(1)-Cl(1)	1.446(9)
O(2)-Cl(1)	1.450(7)
O(3)-Cl(1)	1.454(9)
O(4)-Cl(1)	1.365(7)
O(5)-Cl(2)	1.490(9)
O(6)-Cl(2)	1.375(9)
O(7)-Cl(2)	1.408(9)
O(8)-Cl(2)	1.429(14)
O(9)-H(61)	0.869(17)
O(9)-H(60)	0.88(4)
Cl(1')-O(2')	1.300(12)
Cl(1')-O(1')	1.366(11)
Cl(1')-O(4')	1.411(10)
Cl(1')-O(3')	1.430(9)
Cl(2')-O(6')	1.337(12)
Cl(2')-O(5')	1.338(11)
Cl(2')-O(7')	1.395(9)
Cl(2')-O(8')	1.418(14)

N(1)-C(1)-H(1A)	109.5
N(1)-C(1)-H(1B)	109.5
H(1A)-C(1)-H(1B)	109.5
N(1)-C(1)-H(1C)	109.5
H(1A)-C(1)-H(1C)	109.5
H(1B)-C(1)-H(1C)	109.5
C(3)-C(2)-H(2A)	109.5

C(3)-C(2)-H(2B)	109.5
H(2A)-C(2)-H(2B)	109.5
C(3)-C(2)-H(2C)	109.5
H(2A)-C(2)-H(2C)	109.5
H(2B)-C(2)-H(2C)	109.5
N(1)-C(3)-C(4)	107.2(2)
N(1)-C(3)-C(2)	121.8(2)
C(4)-C(3)-C(2)	131.0(2)
C(3)-C(4)-C(5)	105.3(2)
C(3)-C(4)-H(4)	127.4
C(5)-C(4)-H(4)	127.4
N(2)-C(5)-C(4)	110.8(2)
N(2)-C(5)-C(6)	116.4(2)
C(4)-C(5)-C(6)	132.8(2)
N(3)-C(6)-C(7)	111.3(2)
N(3)-C(6)-C(5)	116.4(2)
C(7)-C(6)-C(5)	132.2(2)
C(8)-C(7)-C(6)	105.1(2)
C(8)-C(7)-H(7)	127.5
C(6)-C(7)-H(7)	127.5
N(4)-C(8)-C(7)	107.0(2)
N(4)-C(8)-C(9)	122.7(2)
C(7)-C(8)-C(9)	130.3(2)
C(8)-C(9)-H(9A)	109.5
C(8)-C(9)-H(9B)	109.5
H(9A)-C(9)-H(9B)	109.5
C(8)-C(9)-H(9C)	109.5
H(9A)-C(9)-H(9C)	109.5
H(9B)-C(9)-H(9C)	109.5
N(4)-C(10)-C(11)	111.6(2)
N(4)-C(10)-H(10A)	109.3
C(11)-C(10)-H(10A)	109.3
N(4)-C(10)-H(10B)	109.3

C(11)-C(10)-H(10B)	109.3
H(10A)-C(10)-H(10B)	108.0
N(5)-C(11)-C(10)	114.08(19)
N(5)-C(11)-H(11A)	108.7
C(10)-C(11)-H(11A)	108.7
N(5)-C(11)-H(11B)	108.7
C(10)-C(11)-H(11B)	108.7
H(11A)-C(11)-H(11B)	107.6
N(5)-C(12)-C(13)	116.7(2)
N(5)-C(12)-H(12A)	108.1
C(13)-C(12)-H(12A)	108.1
N(5)-C(12)-H(12B)	108.1
C(13)-C(12)-H(12B)	108.1
H(12A)-C(12)-H(12B)	107.3
C(14)-C(13)-C(12)	111.1(2)
C(14)-C(13)-H(13A)	109.4
C(12)-C(13)-H(13A)	109.4
C(14)-C(13)-H(13B)	109.4
C(12)-C(13)-H(13B)	109.4
H(13A)-C(13)-H(13B)	108.0
C(13)-C(14)-H(14A)	109.5
C(13)-C(14)-H(14B)	109.5
H(14A)-C(14)-H(14B)	109.5
C(13)-C(14)-H(14C)	109.5
H(14A)-C(14)-H(14C)	109.5
H(14B)-C(14)-H(14C)	109.5
N(5)-C(15)-C(16)	115.7(2)
N(5)-C(15)-H(15A)	108.3
C(16)-C(15)-H(15A)	108.3
N(5)-C(15)-H(15B)	108.3
C(16)-C(15)-H(15B)	108.3
H(15A)-C(15)-H(15B)	107.4
N(6)-C(16)-C(15)	114.6(2)

N(6)-C(16)-H(16A)	108.6
C(15)-C(16)-H(16A)	108.6
N(6)-C(16)-H(16B)	108.6
C(15)-C(16)-H(16B)	108.6
H(16A)-C(16)-H(16B)	107.6
C(18)-C(17)-H(17A)	109.5
C(18)-C(17)-H(17B)	109.5
H(17A)-C(17)-H(17B)	109.5
C(18)-C(17)-H(17C)	109.5
H(17A)-C(17)-H(17C)	109.5
H(17B)-C(17)-H(17C)	109.5
N(6)-C(18)-C(19)	107.0(2)
N(6)-C(18)-C(17)	122.9(2)
C(19)-C(18)-C(17)	130.0(2)
C(18)-C(19)-C(20)	105.6(2)
C(18)-C(19)-H(19)	127.2
C(20)-C(19)-H(19)	127.2
N(7)-C(20)-C(19)	110.8(2)
N(7)-C(20)-C(21)	117.3(2)
C(19)-C(20)-C(21)	131.8(2)
N(8)-C(21)-C(22)	111.5(2)
N(8)-C(21)-C(20)	116.8(2)
C(22)-C(21)-C(20)	131.8(2)
C(23)-C(22)-C(21)	104.8(2)
C(23)-C(22)-H(22)	127.6
C(21)-C(22)-H(22)	127.6
N(9)-C(23)-C(22)	107.3(2)
N(9)-C(23)-C(24)	123.1(2)
C(22)-C(23)-C(24)	129.6(2)
C(23)-C(24)-H(24A)	109.5
C(23)-C(24)-H(24B)	109.5
H(24A)-C(24)-H(24B)	109.5
C(23)-C(24)-H(24C)	109.5

H(24A)-C(24)-H(24C)	109.5
H(24B)-C(24)-H(24C)	109.5
N(9)-C(25)-H(25A)	109.5
N(9)-C(25)-H(25B)	109.5
H(25A)-C(25)-H(25B)	109.5
N(9)-C(25)-H(25C)	109.5
H(25A)-C(25)-H(25C)	109.5
H(25B)-C(25)-H(25C)	109.5
O(10)-C(26)-H(26A)	109.5
O(10)-C(26)-H(26B)	109.5
H(26A)-C(26)-H(26B)	109.5
O(10)-C(26)-H(26C)	109.5
H(26A)-C(26)-H(26C)	109.5
H(26B)-C(26)-H(26C)	109.5
C(3)-N(1)-N(2)	111.24(19)
C(3)-N(1)-C(1)	128.2(2)
N(2)-N(1)-C(1)	120.6(2)
C(5)-N(2)-N(1)	105.43(18)
C(5)-N(2)-Ni(1)	114.27(15)
N(1)-N(2)-Ni(1)	139.87(15)
C(6)-N(3)-N(4)	105.08(18)
C(6)-N(3)-Ni(1)	114.44(15)
N(4)-N(3)-Ni(1)	140.45(15)
N(3)-N(4)-C(8)	111.44(19)
N(3)-N(4)-C(10)	122.03(19)
C(8)-N(4)-C(10)	125.9(2)
C(11)-N(5)-C(12)	110.34(19)
C(11)-N(5)-C(15)	112.2(2)
C(12)-N(5)-C(15)	110.30(18)
N(7)-N(6)-C(18)	110.98(19)
N(7)-N(6)-C(16)	121.60(19)
C(18)-N(6)-C(16)	127.4(2)
C(20)-N(7)-N(6)	105.65(18)

C(20)-N(7)-Ni(1)	113.75(15)
N(6)-N(7)-Ni(1)	140.16(15)
C(21)-N(8)-N(9)	105.04(18)
C(21)-N(8)-Ni(1)	113.76(15)
N(9)-N(8)-Ni(1)	141.03(15)
C(23)-N(9)-N(8)	111.42(19)
C(23)-N(9)-C(25)	127.3(2)
N(8)-N(9)-C(25)	121.10(19)
O(10)-Ni(1)-O(9)	86.83(7)
O(10)-Ni(1)-N(3)	86.24(7)
O(9)-Ni(1)-N(3)	171.31(7)
O(10)-Ni(1)-N(2)	93.42(7)
O(9)-Ni(1)-N(2)	97.10(7)
N(3)-Ni(1)-N(2)	78.10(7)
O(10)-Ni(1)-N(7)	93.44(7)
O(9)-Ni(1)-N(7)	87.09(7)
N(3)-Ni(1)-N(7)	98.54(7)
N(2)-Ni(1)-N(7)	172.15(7)
O(10)-Ni(1)-N(8)	171.62(7)
O(9)-Ni(1)-N(8)	93.39(7)
N(3)-Ni(1)-N(8)	94.23(7)
N(2)-Ni(1)-N(8)	94.87(8)
N(7)-Ni(1)-N(8)	78.22(8)
Ni(1)-O(9)-H(61)	106.5(19)
Ni(1)-O(9)-H(60)	110(2)
H(61)-O(9)-H(60)	100(3)
C(26)-O(10)-Ni(1)	121.97(15)
O(4)-Cl(1)-O(1)	100.2(6)
O(4)-Cl(1)-O(2)	104.0(7)
O(1)-Cl(1)-O(2)	118.7(8)
O(4)-Cl(1)-O(3)	104.2(6)
O(1)-Cl(1)-O(3)	105.3(6)
O(2)-Cl(1)-O(3)	121.3(7)

O(6)-Cl(2)-O(7)	109.5(9)
O(6)-Cl(2)-O(8)	108.8(14)
O(7)-Cl(2)-O(8)	104.2(12)
O(6)-Cl(2)-O(5)	116.3(6)
O(7)-Cl(2)-O(5)	105.1(6)
O(8)-Cl(2)-O(5)	112.2(14)
O(2')-Cl(1')-O(1')	96.4(12)
O(2')-Cl(1')-O(4')	113.8(14)
O(1')-Cl(1')-O(4')	118.8(9)
O(2')-Cl(1')-O(3')	101.5(10)
O(1')-Cl(1')-O(3')	119.4(9)
O(4')-Cl(1')-O(3')	105.5(7)
O(6')-Cl(2')-O(5')	113.7(13)
O(6')-Cl(2')-O(7')	91.3(11)
O(5')-Cl(2')-O(7')	109.1(14)
O(6')-Cl(2')-O(8')	114.5(16)
O(5')-Cl(2')-O(8')	116.9(11)
O(7')-Cl(2')-O(8')	107.9(13)

Table S4. Bond lengths [Å] and angles [°] for **3.**

C(1)-N(2)	1.453(4)
C(1)-H(1A)	0.9800
C(1)-H(1B)	0.9800
C(1)-H(1C)	0.9800
C(2)-C(3)	1.490(5)
C(2)-H(2A)	0.9800
C(2)-H(2B)	0.9800
C(2)-H(2C)	0.9800
C(3)-N(2)	1.355(4)
C(3)-C(4)	1.378(5)

C(4)-C(5)	1.394(4)
C(4)-H(4)	0.9500
C(5)-N(1)	1.341(4)
C(5)-C(6)	1.449(4)
C(6)-N(3)	1.346(4)
C(6)-C(7)	1.392(4)
C(7)-C(8)	1.374(5)
C(7)-H(7)	0.9500
C(8)-N(4)	1.366(4)
C(8)-C(9)	1.487(4)
C(9)-H(9A)	0.9800
C(9)-H(9B)	0.9800
C(9)-H(9C)	0.9800
C(10)-N(4)	1.455(4)
C(10)-C(11)	1.530(4)
C(10)-H(10A)	0.9900
C(10)-H(10B)	0.9900
C(11)-N(5)	1.497(4)
C(11)-H(11A)	0.9900
C(11)-H(11B)	0.9900
C(12)-C(13)	1.521(4)
C(12)-H(12A)	0.9800
C(12)-H(12B)	0.9800
C(12)-H(12C)	0.9800
C(13)-C(14)	1.526(4)
C(13)-H(13A)	0.9900
C(13)-H(13B)	0.9900
C(14)-N(5)	1.491(4)
C(14)-H(14A)	0.9900
C(14)-H(14B)	0.9900
C(15)-N(5)	1.475(4)
C(15)-C(16)	1.525(4)
C(15)-H(15A)	0.9900

C(15)-H(15B)	0.9900
C(16)-N(6)	1.450(4)
C(16)-H(16A)	0.9900
C(16)-H(16B)	0.9900
C(17)-N(6)	1.360(4)
C(17)-C(19)	1.371(5)
C(17)-C(18)	1.490(4)
C(18)-H(18A)	0.9800
C(18)-H(18B)	0.9800
C(18)-H(18C)	0.9800
C(19)-C(20)	1.393(4)
C(19)-H(19)	0.9500
C(20)-N(7)	1.339(4)
C(20)-C(21)	1.452(4)
C(21)-N(8)	1.342(4)
C(21)-C(22)	1.390(4)
C(22)-C(23)	1.369(5)
C(22)-H(22)	0.9500
C(23)-N(9)	1.345(4)
C(23)-C(24)	1.507(5)
C(24)-H(24A)	0.9800
C(24)-H(24B)	0.9800
C(24)-H(24C)	0.9800
C(25)-N(9)	1.458(4)
C(25)-H(25A)	0.9800
C(25)-H(25B)	0.9800
C(25)-H(25C)	0.9800
C(26)-O(5)	1.435(4)
C(26)-H(26A)	0.9800
C(26)-H(26B)	0.9800
C(26)-H(26C)	0.9800
N(1)-N(2)	1.358(3)
N(1)-Co(1)	2.137(3)

N(3)-N(4)	1.360(3)
N(3)-Co(1)	2.159(2)
N(6)-N(7)	1.360(3)
N(7)-Co(1)	2.123(2)
N(8)-N(9)	1.357(3)
N(8)-Co(1)	2.155(3)
Cl(1A)-O(2A)	1.282(18)
Cl(1A)-O(3A)	1.390(11)
Cl(1A)-O(1A)	1.490(15)
Cl(1A)-O(4A)	1.505(13)
Cl(2A)-O(7B)	1.166(16)
Cl(2A)-O(6A)	1.320(14)
Cl(2A)-O(8A)	1.371(10)
Cl(2A)-O(6B)	1.456(14)
Cl(2A)-O(9A)	1.480(14)
Cl(2A)-O(9B)	1.513(16)
Cl(2A)-O(7A)	1.539(9)
Cl(2A)-O(8B)	1.697(14)
O(5)-Co(1)	2.108(2)
O(5)-H(12)	0.84(5)
O(6A)-O(6B)	0.628(17)
O(6A)-Cl(2B)	1.317(15)
O(6A)-O(8B)	1.615(17)
O(7A)-O(7B)	0.897(12)
O(7A)-Cl(2B)	1.461(12)
O(8A)-O(7B)	1.444(13)
O(8A)-Cl(2B)	1.637(11)
O(9A)-O(9B)	0.681(19)
O(9A)-Cl(2B)	1.329(16)
O(10)-Co(1)	2.121(2)
O(10)-H(10)	0.79(4)
O(10)-H(11)	0.81(5)
Cl(1B)-O(1B)	1.34(3)

Cl(1B)-O(4B)	1.375(13)
Cl(1B)-O(3B)	1.495(9)
Cl(1B)-O(2B)	1.55(2)
Cl(2B)-O(7B)	1.249(13)
Cl(2B)-O(9B)	1.431(17)
Cl(2B)-O(8B)	1.434(15)
Cl(2B)-O(6B)	1.553(15)

N(2)-C(1)-H(1A)	109.5
N(2)-C(1)-H(1B)	109.5
H(1A)-C(1)-H(1B)	109.5
N(2)-C(1)-H(1C)	109.5
H(1A)-C(1)-H(1C)	109.5
H(1B)-C(1)-H(1C)	109.5
C(3)-C(2)-H(2A)	109.5
C(3)-C(2)-H(2B)	109.5
H(2A)-C(2)-H(2B)	109.5
C(3)-C(2)-H(2C)	109.5
H(2A)-C(2)-H(2C)	109.5
H(2B)-C(2)-H(2C)	109.5
N(2)-C(3)-C(4)	107.0(3)
N(2)-C(3)-C(2)	123.0(3)
C(4)-C(3)-C(2)	130.0(3)
C(3)-C(4)-C(5)	105.3(3)
C(3)-C(4)-H(4)	127.3
C(5)-C(4)-H(4)	127.3
N(1)-C(5)-C(4)	111.0(3)
N(1)-C(5)-C(6)	117.5(3)
C(4)-C(5)-C(6)	131.5(3)
N(3)-C(6)-C(7)	110.8(3)
N(3)-C(6)-C(5)	117.4(3)
C(7)-C(6)-C(5)	131.8(3)
C(8)-C(7)-C(6)	105.9(3)

C(8)-C(7)-H(7)	127.1
C(6)-C(7)-H(7)	127.1
N(4)-C(8)-C(7)	106.8(3)
N(4)-C(8)-C(9)	122.7(3)
C(7)-C(8)-C(9)	130.4(3)
C(8)-C(9)-H(9A)	109.5
C(8)-C(9)-H(9B)	109.5
H(9A)-C(9)-H(9B)	109.5
C(8)-C(9)-H(9C)	109.5
H(9A)-C(9)-H(9C)	109.5
H(9B)-C(9)-H(9C)	109.5
N(4)-C(10)-C(11)	114.6(3)
N(4)-C(10)-H(10A)	108.6
C(11)-C(10)-H(10A)	108.6
N(4)-C(10)-H(10B)	108.6
C(11)-C(10)-H(10B)	108.6
H(10A)-C(10)-H(10B)	107.6
N(5)-C(11)-C(10)	115.6(3)
N(5)-C(11)-H(11A)	108.4
C(10)-C(11)-H(11A)	108.4
N(5)-C(11)-H(11B)	108.4
C(10)-C(11)-H(11B)	108.4
H(11A)-C(11)-H(11B)	107.4
C(13)-C(12)-H(12A)	109.5
C(13)-C(12)-H(12B)	109.5
H(12A)-C(12)-H(12B)	109.5
C(13)-C(12)-H(12C)	109.5
H(12A)-C(12)-H(12C)	109.5
H(12B)-C(12)-H(12C)	109.5
C(12)-C(13)-C(14)	110.8(3)
C(12)-C(13)-H(13A)	109.5
C(14)-C(13)-H(13A)	109.5
C(12)-C(13)-H(13B)	109.5

C(14)-C(13)-H(13B)	109.5
H(13A)-C(13)-H(13B)	108.1
N(5)-C(14)-C(13)	116.7(3)
N(5)-C(14)-H(14A)	108.1
C(13)-C(14)-H(14A)	108.1
N(5)-C(14)-H(14B)	108.1
C(13)-C(14)-H(14B)	108.1
H(14A)-C(14)-H(14B)	107.3
N(5)-C(15)-C(16)	114.1(3)
N(5)-C(15)-H(15A)	108.7
C(16)-C(15)-H(15A)	108.7
N(5)-C(15)-H(15B)	108.7
C(16)-C(15)-H(15B)	108.7
H(15A)-C(15)-H(15B)	107.6
N(6)-C(16)-C(15)	112.1(3)
N(6)-C(16)-H(16A)	109.2
C(15)-C(16)-H(16A)	109.2
N(6)-C(16)-H(16B)	109.2
C(15)-C(16)-H(16B)	109.2
H(16A)-C(16)-H(16B)	107.9
N(6)-C(17)-C(19)	107.3(3)
N(6)-C(17)-C(18)	122.3(3)
C(19)-C(17)-C(18)	130.4(3)
C(17)-C(18)-H(18A)	109.5
C(17)-C(18)-H(18B)	109.5
H(18A)-C(18)-H(18B)	109.5
C(17)-C(18)-H(18C)	109.5
H(18A)-C(18)-H(18C)	109.5
H(18B)-C(18)-H(18C)	109.5
C(17)-C(19)-C(20)	105.5(3)
C(17)-C(19)-H(19)	127.3
C(20)-C(19)-H(19)	127.3
N(7)-C(20)-C(19)	110.9(3)

N(7)-C(20)-C(21)	117.0(3)
C(19)-C(20)-C(21)	132.0(3)
N(8)-C(21)-C(22)	110.8(3)
N(8)-C(21)-C(20)	116.6(3)
C(22)-C(21)-C(20)	132.6(3)
C(23)-C(22)-C(21)	105.4(3)
C(23)-C(22)-H(22)	127.3
C(21)-C(22)-H(22)	127.3
N(9)-C(23)-C(22)	107.4(3)
N(9)-C(23)-C(24)	121.5(3)
C(22)-C(23)-C(24)	131.1(3)
C(23)-C(24)-H(24A)	109.5
C(23)-C(24)-H(24B)	109.5
H(24A)-C(24)-H(24B)	109.5
C(23)-C(24)-H(24C)	109.5
H(24A)-C(24)-H(24C)	109.5
H(24B)-C(24)-H(24C)	109.5
N(9)-C(25)-H(25A)	109.5
N(9)-C(25)-H(25B)	109.5
H(25A)-C(25)-H(25B)	109.5
N(9)-C(25)-H(25C)	109.5
H(25A)-C(25)-H(25C)	109.5
H(25B)-C(25)-H(25C)	109.5
O(5)-C(26)-H(26A)	109.5
O(5)-C(26)-H(26B)	109.5
H(26A)-C(26)-H(26B)	109.5
O(5)-C(26)-H(26C)	109.5
H(26A)-C(26)-H(26C)	109.5
H(26B)-C(26)-H(26C)	109.5
C(5)-N(1)-N(2)	105.2(2)
C(5)-N(1)-Co(1)	114.1(2)
N(2)-N(1)-Co(1)	140.5(2)
C(3)-N(2)-N(1)	111.4(3)

C(3)-N(2)-C(1)	127.6(3)
N(1)-N(2)-C(1)	121.0(2)
C(6)-N(3)-N(4)	105.3(2)
C(6)-N(3)-Co(1)	113.29(19)
N(4)-N(3)-Co(1)	140.91(19)
N(3)-N(4)-C(8)	111.1(2)
N(3)-N(4)-C(10)	121.2(2)
C(8)-N(4)-C(10)	127.6(3)
C(15)-N(5)-C(14)	110.3(2)
C(15)-N(5)-C(11)	112.2(3)
C(14)-N(5)-C(11)	110.5(2)
C(17)-N(6)-N(7)	110.8(2)
C(17)-N(6)-C(16)	126.8(3)
N(7)-N(6)-C(16)	121.9(2)
C(20)-N(7)-N(6)	105.5(2)
C(20)-N(7)-Co(1)	114.97(19)
N(6)-N(7)-Co(1)	139.52(19)
C(21)-N(8)-N(9)	105.0(2)
C(21)-N(8)-Co(1)	113.85(19)
N(9)-N(8)-Co(1)	140.44(19)
C(23)-N(9)-N(8)	111.3(3)
C(23)-N(9)-C(25)	128.5(3)
N(8)-N(9)-C(25)	120.1(3)
O(2A)-Cl(1A)-O(3A)	116.8(12)
O(2A)-Cl(1A)-O(1A)	100.3(12)
O(3A)-Cl(1A)-O(1A)	121.9(8)
O(2A)-Cl(1A)-O(4A)	106.0(12)
O(3A)-Cl(1A)-O(4A)	102.3(7)
O(1A)-Cl(1A)-O(4A)	108.7(7)
O(7B)-Cl(2A)-O(6A)	124.4(10)
O(7B)-Cl(2A)-O(8A)	68.9(8)
O(6A)-Cl(2A)-O(8A)	121.0(9)
O(7B)-Cl(2A)-O(6B)	131.4(10)

O(6A)-Cl(2A)-O(6B)	25.5(8)
O(8A)-Cl(2A)-O(6B)	98.4(7)
O(7B)-Cl(2A)-O(9A)	103.6(9)
O(6A)-Cl(2A)-O(9A)	120.6(10)
O(8A)-Cl(2A)-O(9A)	106.8(7)
O(6B)-Cl(2A)-O(9A)	124.8(9)
O(7B)-Cl(2A)-O(9B)	125.7(9)
O(6A)-Cl(2A)-O(9B)	107.2(9)
O(8A)-Cl(2A)-O(9B)	99.9(8)
O(6B)-Cl(2A)-O(9B)	102.4(7)
O(9A)-Cl(2A)-O(9B)	26.3(7)
O(7B)-Cl(2A)-O(7A)	35.4(6)
O(6A)-Cl(2A)-O(7A)	103.1(8)
O(8A)-Cl(2A)-O(7A)	104.2(6)
O(6B)-Cl(2A)-O(7A)	123.7(8)
O(9A)-Cl(2A)-O(7A)	96.7(8)
O(9B)-Cl(2A)-O(7A)	122.8(8)
O(7B)-Cl(2A)-O(8B)	103.7(9)
O(6A)-Cl(2A)-O(8B)	63.3(8)
O(8A)-Cl(2A)-O(8B)	172.5(7)
O(6B)-Cl(2A)-O(8B)	87.0(7)
O(9A)-Cl(2A)-O(8B)	73.9(7)
O(9B)-Cl(2A)-O(8B)	83.9(8)
O(7A)-Cl(2A)-O(8B)	68.4(6)
C(26)-O(5)-Co(1)	122.02(19)
C(26)-O(5)-H(12)	107(3)
Co(1)-O(5)-H(12)	106(3)
O(6B)-O(6A)-Cl(2B)	100(2)
O(6B)-O(6A)-Cl(2A)	89(2)
Cl(2B)-O(6A)-Cl(2A)	12.5(8)
O(6B)-O(6A)-O(8B)	149(3)
Cl(2B)-O(6A)-O(8B)	57.5(8)
Cl(2A)-O(6A)-O(8B)	69.8(8)

O(7B)-O(7A)-Cl(2B)	58.3(9)
O(7B)-O(7A)-Cl(2A)	48.9(10)
Cl(2B)-O(7A)-Cl(2A)	10.6(7)
Cl(2A)-O(8A)-O(7B)	48.8(7)
Cl(2A)-O(8A)-Cl(2B)	4.1(7)
O(7B)-O(8A)-Cl(2B)	47.3(5)
O(9B)-O(9A)-Cl(2B)	84(2)
O(9B)-O(9A)-Cl(2A)	80(2)
Cl(2B)-O(9A)-Cl(2A)	10.0(8)
Co(1)-O(10)-H(10)	116(3)
Co(1)-O(10)-H(11)	113(3)
H(10)-O(10)-H(11)	108(4)
O(5)-Co(1)-O(10)	86.51(9)
O(5)-Co(1)-N(7)	85.55(9)
O(10)-Co(1)-N(7)	170.05(9)
O(5)-Co(1)-N(1)	170.37(9)
O(10)-Co(1)-N(1)	93.89(9)
N(7)-Co(1)-N(1)	94.95(9)
O(5)-Co(1)-N(8)	93.70(9)
O(10)-Co(1)-N(8)	97.59(9)
N(7)-Co(1)-N(8)	76.97(9)
N(1)-Co(1)-N(8)	95.78(10)
O(5)-Co(1)-N(3)	92.91(9)
O(10)-Co(1)-N(3)	86.58(10)
N(7)-Co(1)-N(3)	99.79(9)
N(1)-Co(1)-N(3)	77.53(10)
N(8)-Co(1)-N(3)	172.39(10)
O(1B)-Cl(1B)-O(4B)	97.7(12)
O(1B)-Cl(1B)-O(3B)	89.8(12)
O(4B)-Cl(1B)-O(3B)	116.4(6)
O(1B)-Cl(1B)-O(2B)	115.5(15)
O(4B)-Cl(1B)-O(2B)	121.7(10)
O(3B)-Cl(1B)-O(2B)	110.4(9)

O(7B)-Cl(2B)-O(6A)	118.0(12)
O(7B)-Cl(2B)-O(9A)	108.1(11)
O(6A)-Cl(2B)-O(9A)	133.8(12)
O(7B)-Cl(2B)-O(9B)	125.9(12)
O(6A)-Cl(2B)-O(9B)	112.4(10)
O(9A)-Cl(2B)-O(9B)	28.3(8)
O(7B)-Cl(2B)-O(8B)	115.7(10)
O(6A)-Cl(2B)-O(8B)	71.8(9)
O(9A)-Cl(2B)-O(8B)	87.8(9)
O(9B)-Cl(2B)-O(8B)	97.2(9)
O(7B)-Cl(2B)-O(7A)	37.6(7)
O(6A)-Cl(2B)-O(7A)	107.7(10)
O(9A)-Cl(2B)-O(7A)	107.8(10)
O(9B)-Cl(2B)-O(7A)	135.8(9)
O(8B)-Cl(2B)-O(7A)	78.1(7)
O(7B)-Cl(2B)-O(6B)	116.8(10)
O(6A)-Cl(2B)-O(6B)	23.5(8)
O(9A)-Cl(2B)-O(6B)	128.9(11)
O(9B)-Cl(2B)-O(6B)	101.6(9)
O(8B)-Cl(2B)-O(6B)	93.5(9)
O(7A)-Cl(2B)-O(6B)	122.4(9)
O(7B)-Cl(2B)-O(8A)	58.2(7)
O(6A)-Cl(2B)-O(8A)	104.4(9)
O(9A)-Cl(2B)-O(8A)	100.5(8)
O(9B)-Cl(2B)-O(8A)	91.9(8)
O(8B)-Cl(2B)-O(8A)	170.9(9)
O(7A)-Cl(2B)-O(8A)	95.6(7)
O(6B)-Cl(2B)-O(8A)	84.3(7)
Cl(2B)-O(8B)-O(6A)	50.7(7)
Cl(2B)-O(8B)-Cl(2A)	4.2(7)
O(6A)-O(8B)-Cl(2A)	46.9(6)
O(7A)-O(7B)-Cl(2A)	95.7(13)
O(7A)-O(7B)-Cl(2B)	84.1(13)

Cl(2A)-O(7B)-Cl(2B)	13.0(9)
O(7A)-O(7B)-O(8A)	157.4(15)
Cl(2A)-O(7B)-O(8A)	62.3(8)
Cl(2B)-O(7B)-O(8A)	74.5(8)
O(9A)-O(9B)-Cl(2B)	68(2)
O(9A)-O(9B)-Cl(2A)	74(2)
Cl(2B)-O(9B)-Cl(2A)	10.7(7)
O(6A)-O(6B)-Cl(2A)	65.0(19)
O(6A)-O(6B)-Cl(2B)	56.6(19)
Cl(2A)-O(6B)-Cl(2B)	10.3(7)

Table S5. Bond lengths [Å] and angles [°] for **4.**

C(1)-N(9)	1.455(3)
C(1)-H(1A)	0.9800
C(1)-H(1B)	0.9800
C(1)-H(1C)	0.9800
C(2)-C(3)	1.486(4)
C(2)-H(2A)	0.9800
C(2)-H(2B)	0.9800
C(2)-H(2C)	0.9800
C(3)-N(9)	1.359(3)
C(3)-C(4)	1.383(4)
C(4)-C(5)	1.395(4)
C(4)-H(4)	0.9500
C(5)-N(8)	1.342(3)
C(5)-C(6)	1.463(4)
C(6)-N(7)	1.340(3)
C(6)-C(7)	1.397(3)
C(7)-C(8)	1.379(3)
C(7)-H(7)	0.9500

C(8)-N(6)	1.362(3)
C(8)-C(9)	1.493(4)
C(9)-H(9A)	0.9800
C(9)-H(9B)	0.9800
C(9)-H(9C)	0.9800
C(10)-N(6)	1.455(3)
C(10)-C(11)	1.527(4)
C(10)-H(10A)	0.9900
C(10)-H(10B)	0.9900
C(11)-N(5)	1.458(3)
C(11)-H(11A)	0.9900
C(11)-H(11B)	0.9900
C(12)-N(5)	1.474(4)
C(12)-C(13)	1.539(5)
C(12)-H(12A)	0.9900
C(12)-H(12B)	0.9900
C(13)-C(14)	1.545(7)
C(13)-H(13A)	0.9900
C(13)-H(13B)	0.9900
C(14)-H(14A)	0.9800
C(14)-H(14B)	0.9800
C(14)-H(14C)	0.9800
C(15)-N(5)	1.459(4)
C(15)-C(16)	1.541(4)
C(15)-H(15A)	0.9900
C(15)-H(15B)	0.9900
C(16)-N(4)	1.459(3)
C(16)-H(16A)	0.9900
C(16)-H(16B)	0.9900
C(17)-C(18)	1.492(4)
C(17)-H(17A)	0.9800
C(17)-H(17B)	0.9800
C(17)-H(17C)	0.9800

C(18)-N(4)	1.363(3)
C(18)-C(19)	1.368(4)
C(19)-C(20)	1.401(4)
C(19)-H(19)	0.9500
C(20)-N(3)	1.343(3)
C(20)-C(21)	1.448(4)
C(21)-N(1)	1.342(3)
C(21)-C(22)	1.400(4)
C(22)-C(23)	1.381(4)
C(22)-H(22)	0.9500
C(23)-N(2)	1.356(4)
C(23)-C(24)	1.506(4)
C(24)-H(24A)	0.9800
C(24)-H(24B)	0.9800
C(24)-H(24C)	0.9800
C(25)-N(2)	1.449(4)
C(25)-H(25A)	0.9800
C(25)-H(25B)	0.9800
C(25)-H(25C)	0.9800
C(26)-N(10)	1.170(4)
C(26)-S(2)	1.630(3)
C(27)-N(11)	1.160(3)
C(27)-S(1)	1.630(3)
N(1)-N(2)	1.362(3)
N(1)-Fe(1)	2.226(2)
N(3)-N(4)	1.367(3)
N(3)-Fe(1)	2.304(2)
N(6)-N(7)	1.365(3)
N(7)-Fe(1)	2.235(2)
N(8)-N(9)	1.363(3)
N(8)-Fe(1)	2.274(2)
N(10)-Fe(1)	2.085(3)
N(11)-Fe(1)	2.099(3)

N(9)-C(1)-H(1A)	109.5
N(9)-C(1)-H(1B)	109.5
H(1A)-C(1)-H(1B)	109.5
N(9)-C(1)-H(1C)	109.5
H(1A)-C(1)-H(1C)	109.5
H(1B)-C(1)-H(1C)	109.5
C(3)-C(2)-H(2A)	109.5
C(3)-C(2)-H(2B)	109.5
H(2A)-C(2)-H(2B)	109.5
C(3)-C(2)-H(2C)	109.5
H(2A)-C(2)-H(2C)	109.5
H(2B)-C(2)-H(2C)	109.5
N(9)-C(3)-C(4)	106.3(2)
N(9)-C(3)-C(2)	122.7(3)
C(4)-C(3)-C(2)	131.0(3)
C(3)-C(4)-C(5)	105.7(2)
C(3)-C(4)-H(4)	127.1
C(5)-C(4)-H(4)	127.1
N(8)-C(5)-C(4)	111.2(2)
N(8)-C(5)-C(6)	117.8(2)
C(4)-C(5)-C(6)	131.1(2)
N(7)-C(6)-C(7)	111.2(2)
N(7)-C(6)-C(5)	117.6(2)
C(7)-C(6)-C(5)	131.2(2)
C(8)-C(7)-C(6)	105.6(2)
C(8)-C(7)-H(7)	127.2
C(6)-C(7)-H(7)	127.2
N(6)-C(8)-C(7)	106.6(2)
N(6)-C(8)-C(9)	123.2(2)
C(7)-C(8)-C(9)	130.1(2)
C(8)-C(9)-H(9A)	109.5
C(8)-C(9)-H(9B)	109.5

H(9A)-C(9)-H(9B)	109.5
C(8)-C(9)-H(9C)	109.5
H(9A)-C(9)-H(9C)	109.5
H(9B)-C(9)-H(9C)	109.5
N(6)-C(10)-C(11)	111.9(2)
N(6)-C(10)-H(10A)	109.2
C(11)-C(10)-H(10A)	109.2
N(6)-C(10)-H(10B)	109.2
C(11)-C(10)-H(10B)	109.2
H(10A)-C(10)-H(10B)	107.9
N(5)-C(11)-C(10)	109.9(2)
N(5)-C(11)-H(11A)	109.7
C(10)-C(11)-H(11A)	109.7
N(5)-C(11)-H(11B)	109.7
C(10)-C(11)-H(11B)	109.7
H(11A)-C(11)-H(11B)	108.2
N(5)-C(12)-C(13)	113.4(3)
N(5)-C(12)-H(12A)	108.9
C(13)-C(12)-H(12A)	108.9
N(5)-C(12)-H(12B)	108.9
C(13)-C(12)-H(12B)	108.9
H(12A)-C(12)-H(12B)	107.7
C(12)-C(13)-C(14)	111.9(3)
C(12)-C(13)-H(13A)	109.2
C(14)-C(13)-H(13A)	109.2
C(12)-C(13)-H(13B)	109.2
C(14)-C(13)-H(13B)	109.2
H(13A)-C(13)-H(13B)	107.9
C(13)-C(14)-H(14A)	109.5
C(13)-C(14)-H(14B)	109.5
H(14A)-C(14)-H(14B)	109.5
C(13)-C(14)-H(14C)	109.5
H(14A)-C(14)-H(14C)	109.5

H(14B)-C(14)-H(14C)	109.5
N(5)-C(15)-C(16)	113.1(2)
N(5)-C(15)-H(15A)	109.0
C(16)-C(15)-H(15A)	109.0
N(5)-C(15)-H(15B)	109.0
C(16)-C(15)-H(15B)	109.0
H(15A)-C(15)-H(15B)	107.8
N(4)-C(16)-C(15)	113.1(2)
N(4)-C(16)-H(16A)	109.0
C(15)-C(16)-H(16A)	109.0
N(4)-C(16)-H(16B)	109.0
C(15)-C(16)-H(16B)	109.0
H(16A)-C(16)-H(16B)	107.8
C(18)-C(17)-H(17A)	109.5
C(18)-C(17)-H(17B)	109.5
H(17A)-C(17)-H(17B)	109.5
C(18)-C(17)-H(17C)	109.5
H(17A)-C(17)-H(17C)	109.5
H(17B)-C(17)-H(17C)	109.5
N(4)-C(18)-C(19)	107.1(2)
N(4)-C(18)-C(17)	122.8(3)
C(19)-C(18)-C(17)	130.1(3)
C(18)-C(19)-C(20)	105.8(3)
C(18)-C(19)-H(19)	127.1
C(20)-C(19)-H(19)	127.1
N(3)-C(20)-C(19)	111.0(2)
N(3)-C(20)-C(21)	119.1(2)
C(19)-C(20)-C(21)	130.0(3)
N(1)-C(21)-C(22)	110.8(2)
N(1)-C(21)-C(20)	118.4(2)
C(22)-C(21)-C(20)	130.9(3)
C(23)-C(22)-C(21)	105.5(3)
C(23)-C(22)-H(22)	127.2

C(21)-C(22)-H(22)	127.2
N(2)-C(23)-C(22)	106.9(2)
N(2)-C(23)-C(24)	122.3(3)
C(22)-C(23)-C(24)	130.9(3)
C(23)-C(24)-H(24A)	109.5
C(23)-C(24)-H(24B)	109.5
H(24A)-C(24)-H(24B)	109.5
C(23)-C(24)-H(24C)	109.5
H(24A)-C(24)-H(24C)	109.5
H(24B)-C(24)-H(24C)	109.5
N(2)-C(25)-H(25A)	109.5
N(2)-C(25)-H(25B)	109.5
H(25A)-C(25)-H(25B)	109.5
N(2)-C(25)-H(25C)	109.5
H(25A)-C(25)-H(25C)	109.5
H(25B)-C(25)-H(25C)	109.5
N(10)-C(26)-S(2)	179.2(3)
N(11)-C(27)-S(1)	179.8(3)
C(21)-N(1)-N(2)	105.3(2)
C(21)-N(1)-Fe(1)	115.00(18)
N(2)-N(1)-Fe(1)	138.72(18)
C(23)-N(2)-N(1)	111.5(2)
C(23)-N(2)-C(25)	128.2(2)
N(1)-N(2)-C(25)	120.3(2)
C(20)-N(3)-N(4)	104.9(2)
C(20)-N(3)-Fe(1)	112.10(17)
N(4)-N(3)-Fe(1)	142.24(17)
C(18)-N(4)-N(3)	111.3(2)
C(18)-N(4)-C(16)	128.1(2)
N(3)-N(4)-C(16)	120.2(2)
C(11)-N(5)-C(15)	113.0(2)
C(11)-N(5)-C(12)	113.0(2)
C(15)-N(5)-C(12)	112.3(2)

C(8)-N(6)-N(7)	111.5(2)
C(8)-N(6)-C(10)	129.3(2)
N(7)-N(6)-C(10)	119.17(19)
C(6)-N(7)-N(6)	105.02(19)
C(6)-N(7)-Fe(1)	116.06(16)
N(6)-N(7)-Fe(1)	138.91(16)
C(5)-N(8)-N(9)	104.8(2)
C(5)-N(8)-Fe(1)	114.52(16)
N(9)-N(8)-Fe(1)	140.38(16)
C(3)-N(9)-N(8)	111.9(2)
C(3)-N(9)-C(1)	127.2(2)
N(8)-N(9)-C(1)	120.9(2)
C(26)-N(10)-Fe(1)	169.3(3)
C(27)-N(11)-Fe(1)	168.1(2)
N(10)-Fe(1)-N(11)	96.71(10)
N(10)-Fe(1)-N(1)	88.41(10)
N(11)-Fe(1)-N(1)	168.85(9)
N(10)-Fe(1)-N(7)	168.02(9)
N(11)-Fe(1)-N(7)	89.46(9)
N(1)-Fe(1)-N(7)	87.36(8)
N(10)-Fe(1)-N(8)	95.58(9)
N(11)-Fe(1)-N(8)	92.04(9)
N(1)-Fe(1)-N(8)	97.33(8)
N(7)-Fe(1)-N(8)	73.88(8)
N(10)-Fe(1)-N(3)	90.67(9)
N(11)-Fe(1)-N(3)	95.60(9)
N(1)-Fe(1)-N(3)	74.37(8)
N(7)-Fe(1)-N(3)	98.97(8)
N(8)-Fe(1)-N(3)	169.51(8)

Fig. S1 FT-IR spectroscopy comparison plot of **L** with **1** - **4**.

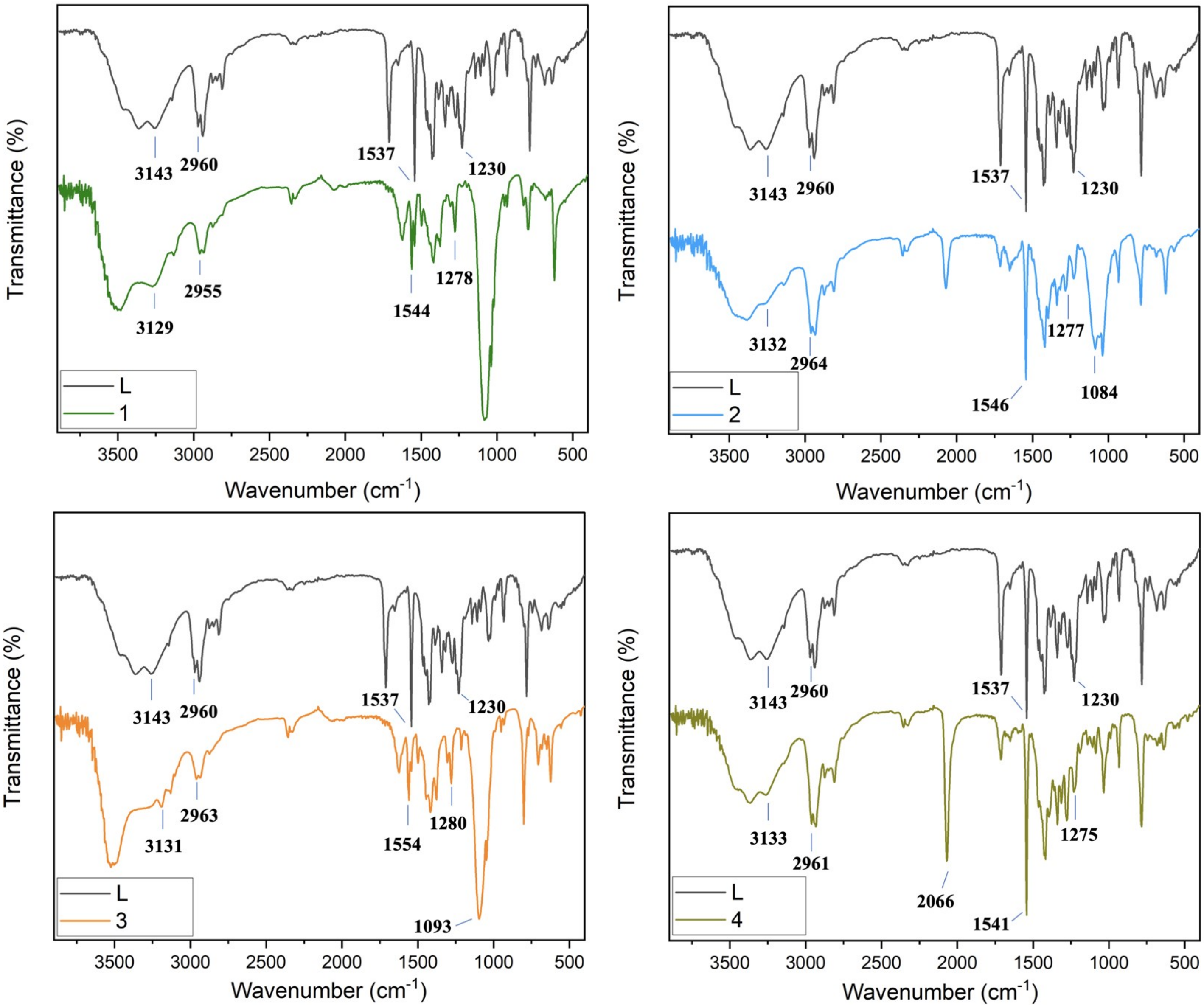

Fig. S2 Diffuse reflectance spectroscopy comparison plot of **L** with **1** - **4**.

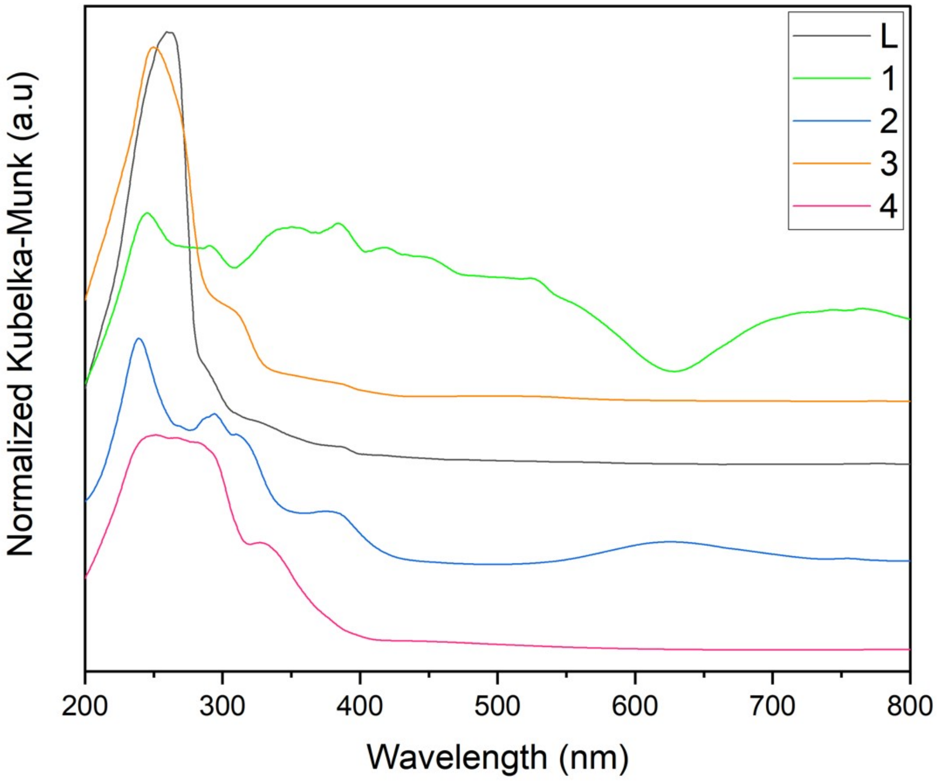

1. ^{1}H-NMR:

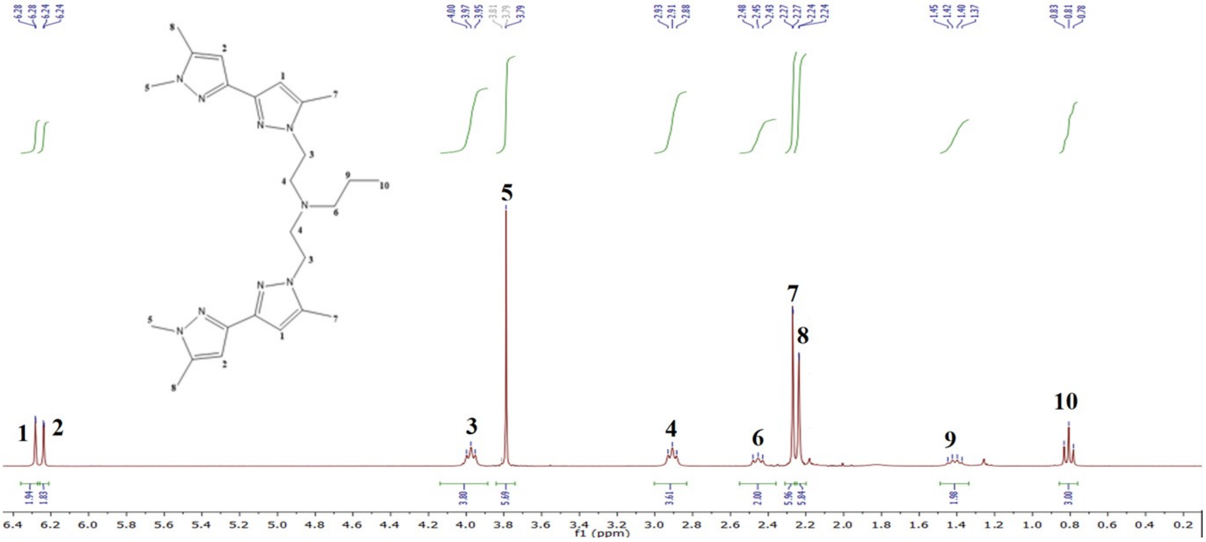

3. ^{13}C-NMR:

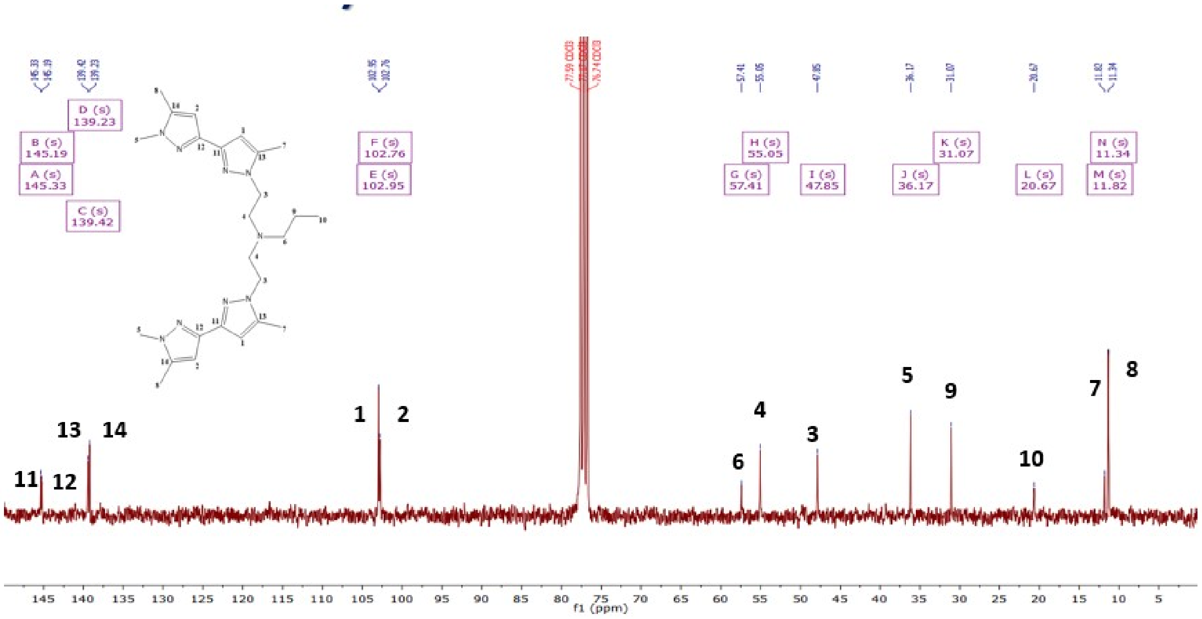

4. HRMS of L:

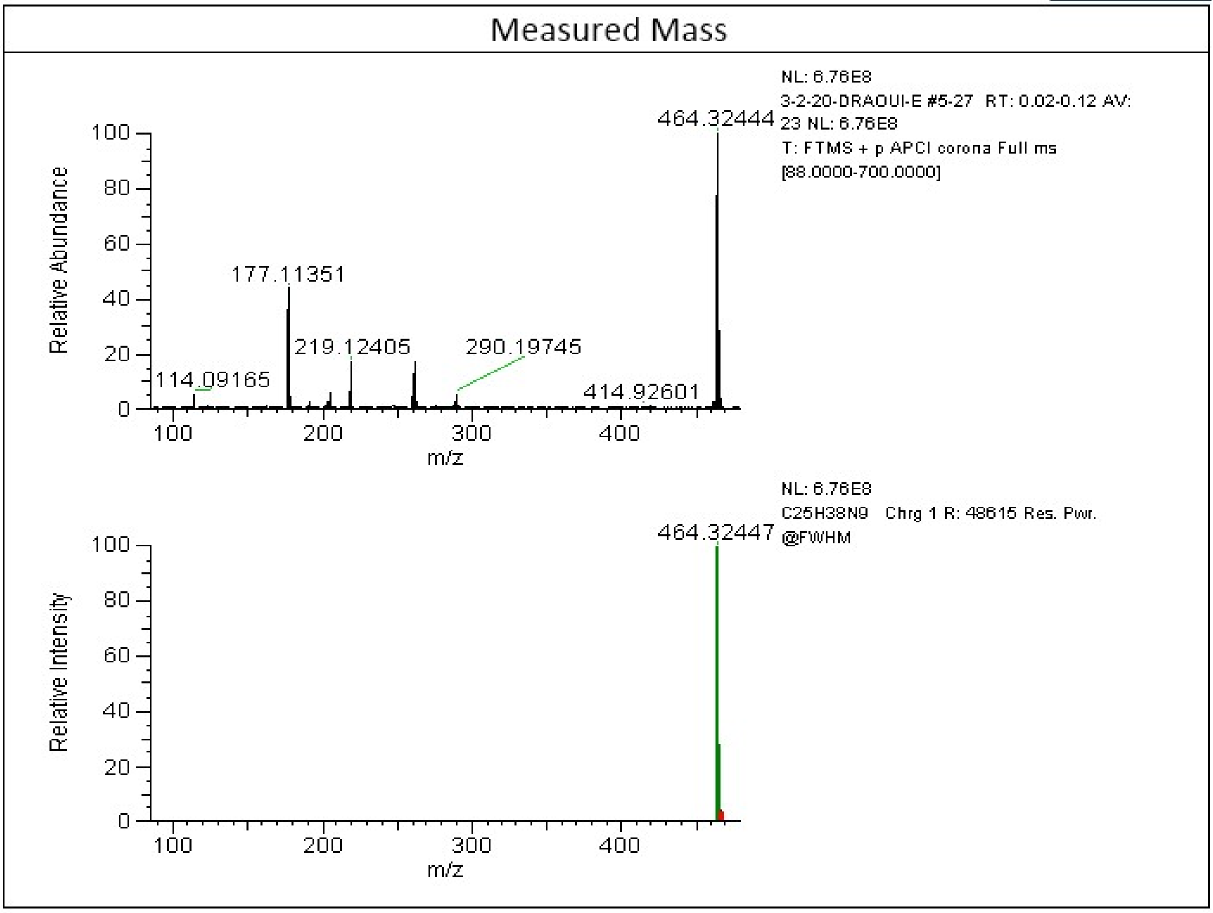

5. Elemental composition of L:

Peak Mass	Display Formula	MS Cov. [%]	Delta [ppm]	Theo. mass
464.32444	$C_{25}H_{38}N_9$	99.98	-0.005	464.32447

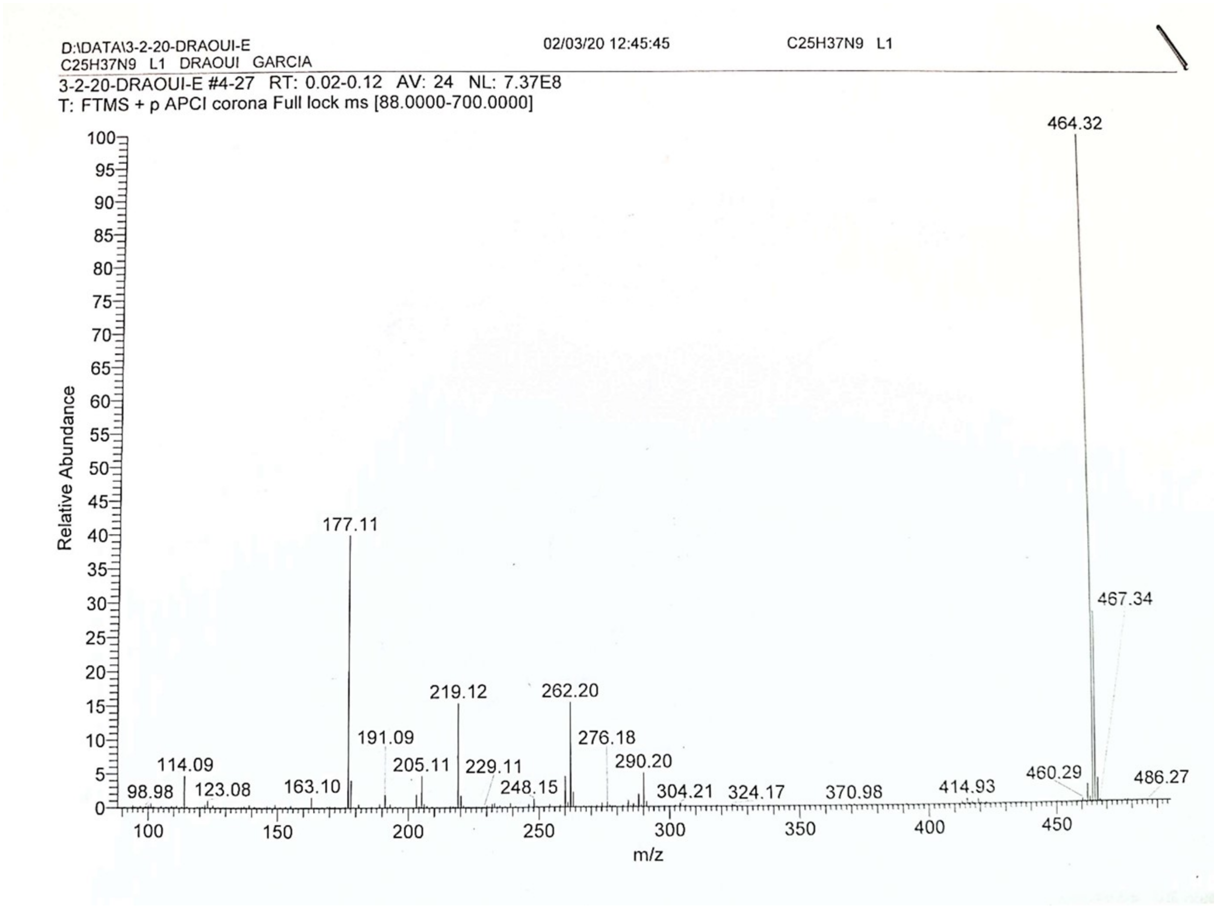
D:\DATA\3-2-20-DRAOUI-E
02/03/20 12:45:45
C25H37N9 L1
C25H37N9 L1 DRAOUI GARCIA
3-2-20-DRAOUI-E #4-27 RT: 0.02-0.12 AV: 24 NL: 7.37E8
T: FTMS + p APCI corona Full lock ms [88.0000-700.0000]
Relative Abundance
m/z
464.32
467.34
177.11
219.12
262.20
191.09
276.18
205.11
229.11
290.20
114.09
98.98
123.08
163.10
248.15
304.21
324.17
370.98
414.93
460.29
486.27

6. HRMS of *Cu_2LCl_4* :

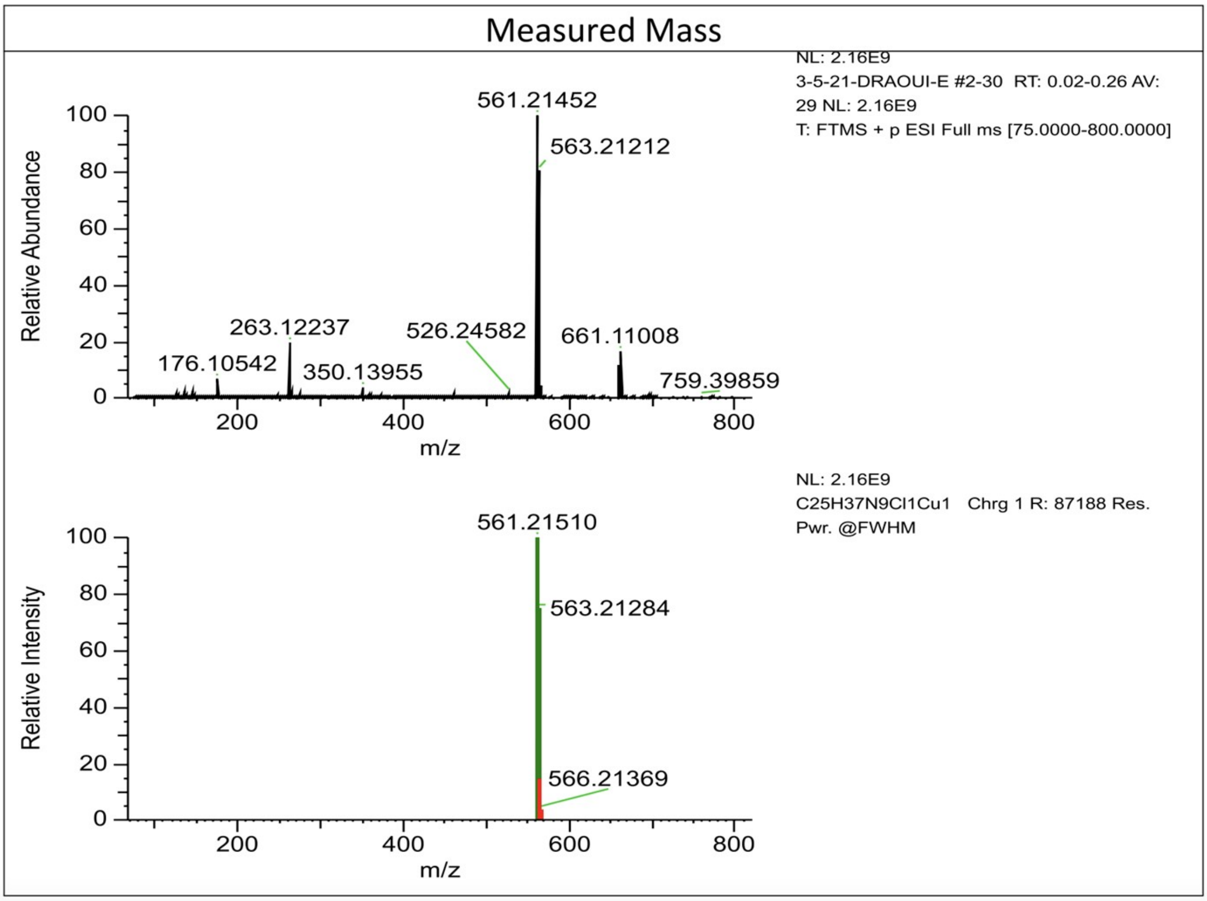

7. Elemental composition of *Cu_2LCl_4*:

Peak Mass	Display Formula	MS Cov.[%]	Delta[ppm]	Theo.mass
561.21452	$C_{25}H_{37}N_9{}^{35}Cl^{63}Cu$	99.95	-1.03	561.21510
659.11254	$C_{25}H_{37}N_9{}^{35}Cl_2{}^{63}Cu_2$	99.89	-1.53	659.11355
263.12237	$C_{25}H_{37}N_9{}^{63}Cu$	97.32	-1.80	263.12285

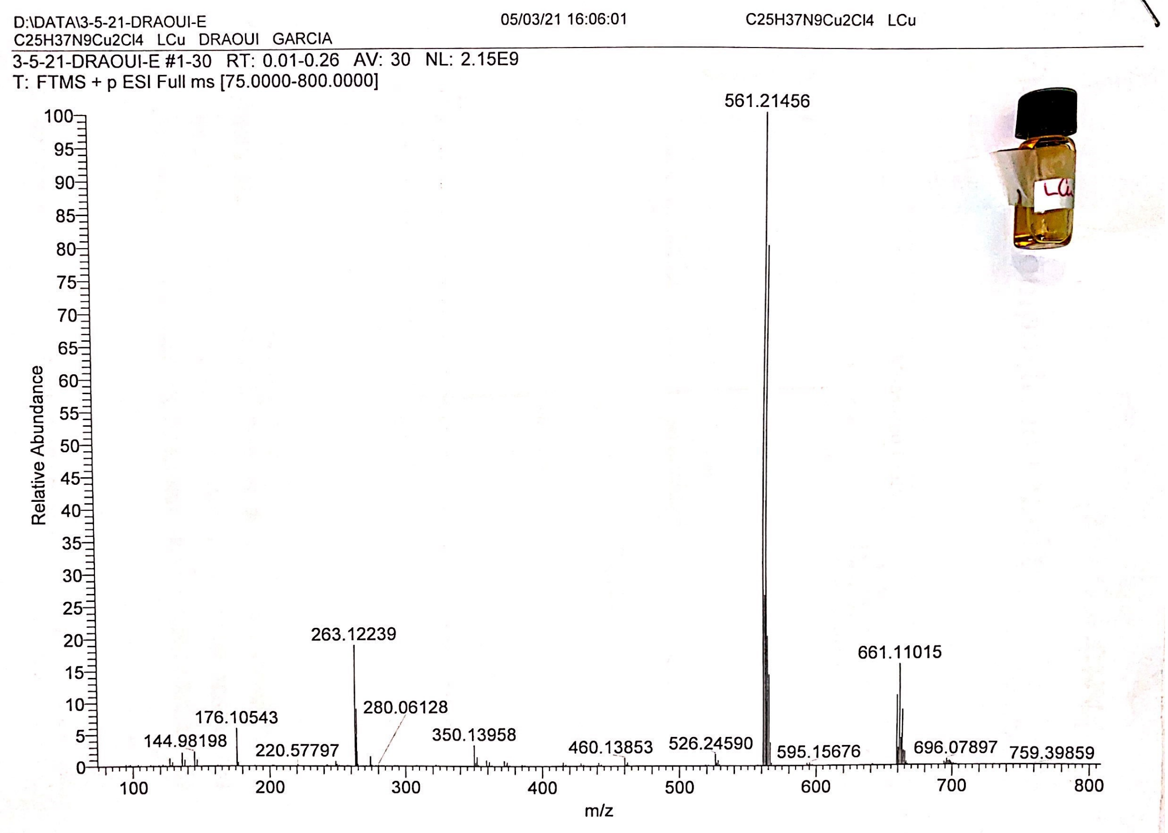
D:\DATA\3-5-21-DRAOUI-E
05/03/21 16:06:01
C25H37N9Cu2Cl4 LCu
C25H37N9Cu2Cl4 LCu DRAOUI GARCIA
3-5-21-DRAOUI-E #1-30 RT: 0.01-0.26 AV: 30 NL: 2.15E9
T: FTMS + p ESI Full ms [75.0000-800.0000]
Relative Abundance
m/z
561.21456
263.12239
661.11015
280.06128
176.10543
350.13958
144.98198
220.57797
460.13853
526.24590
595.15676
696.07897
759.39859

8. **HRMS of** $NiL(CH_3OH)(H_2O)$**:**

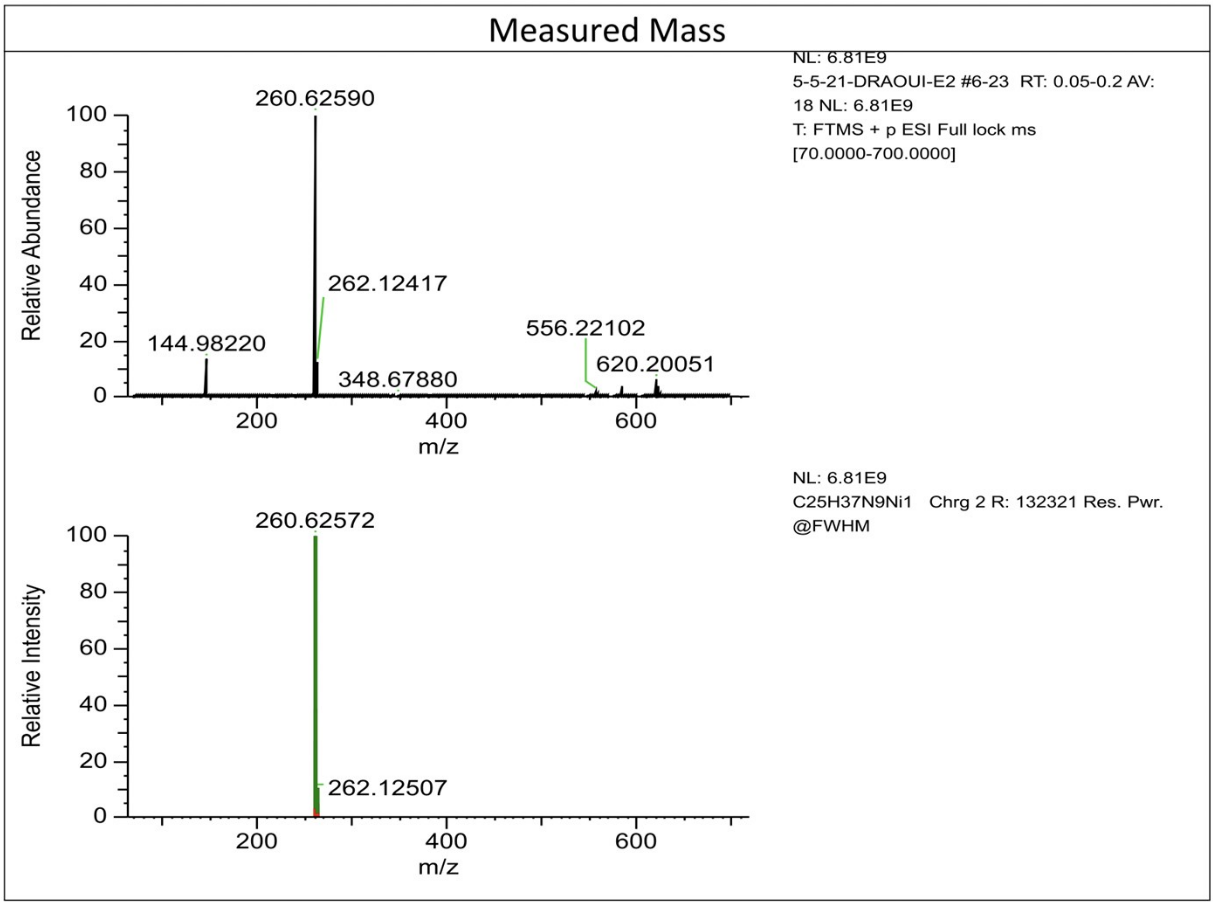

9. **Elemental composition of** $NiL(CH_3OH)(H_2O)$**:**

Peak Mass	Display Formula	MS Cov.[%]	Delta[ppm]	Theo.mass
260.62590	$C_{25}H_{37}N_9{}^{58}Ni$	97.97	0.69	260.62572

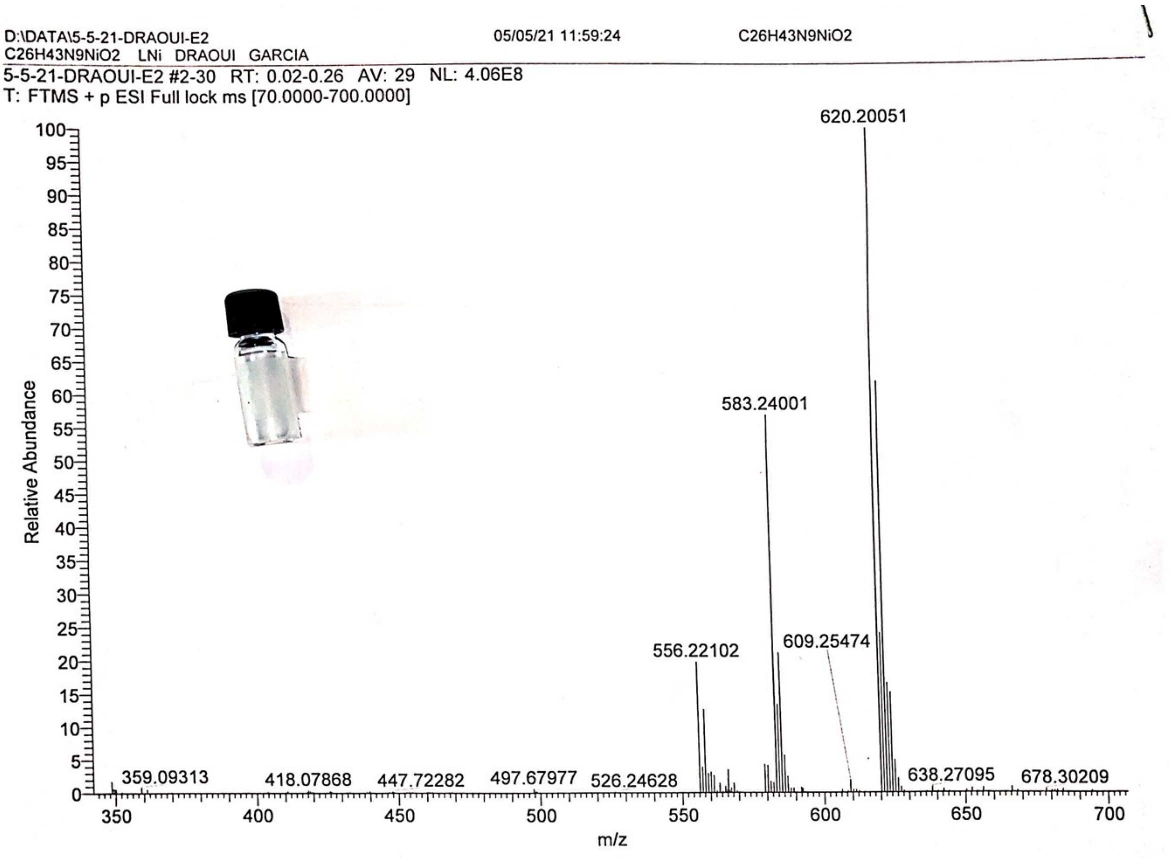
D:\DATA\5-5-21-DRAOUI-E2
05/05/21 11:59:24
C26H43N9NiO2
C26H43N9NiO2 LNi DRAOUI GARCIA
5-5-21-DRAOUI-E2 #2-30 RT: 0.02-0.26 AV: 29 NL: 4.06E8
T: FTMS + p ESI Full lock ms [70.0000-700.0000]
620.20051
583.24001
556.22102
609.25474
359.09313
418.07868
447.72282
497.67977
526.24628
638.27095
678.30209
Relative Abundance
m/z

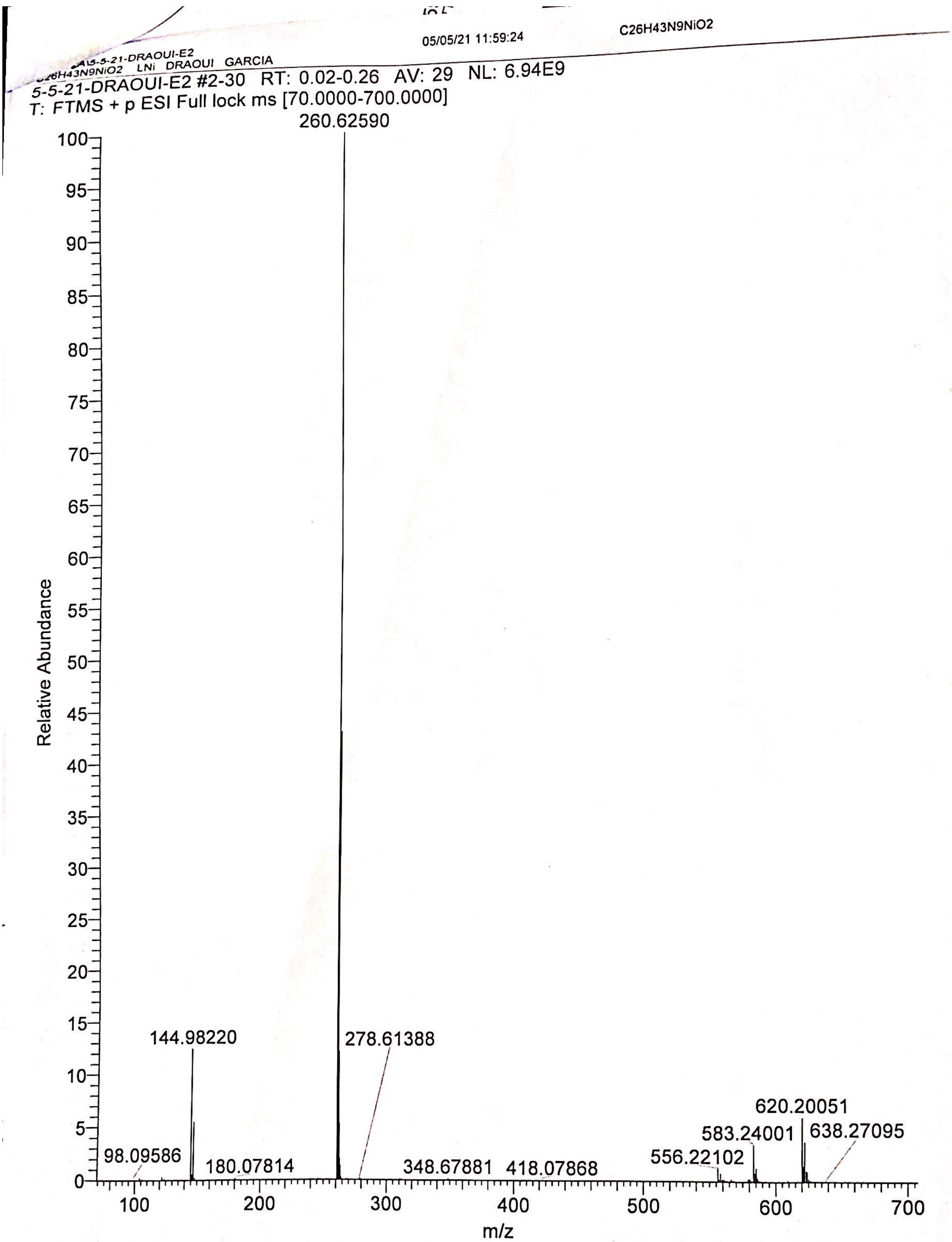
05/05/21 11:59:24
C26H43N9NiO2
C26H43N9NiO2 LNi DRAOUI GARCIA
5-5-21-DRAOUI-E2 #2-30 RT: 0.02-0.26 AV: 29 NL: 6.94E9
T: FTMS + p ESI Full lock ms [70.0000-700.0000]
260.62590
144.98220
278.61388
620.20051
583.24001
638.27095
556.22102
98.09586
180.07814
348.67881
418.07868
Relative Abundance
m/z

10. HRMS of [FeL(NCS)$_2$]:

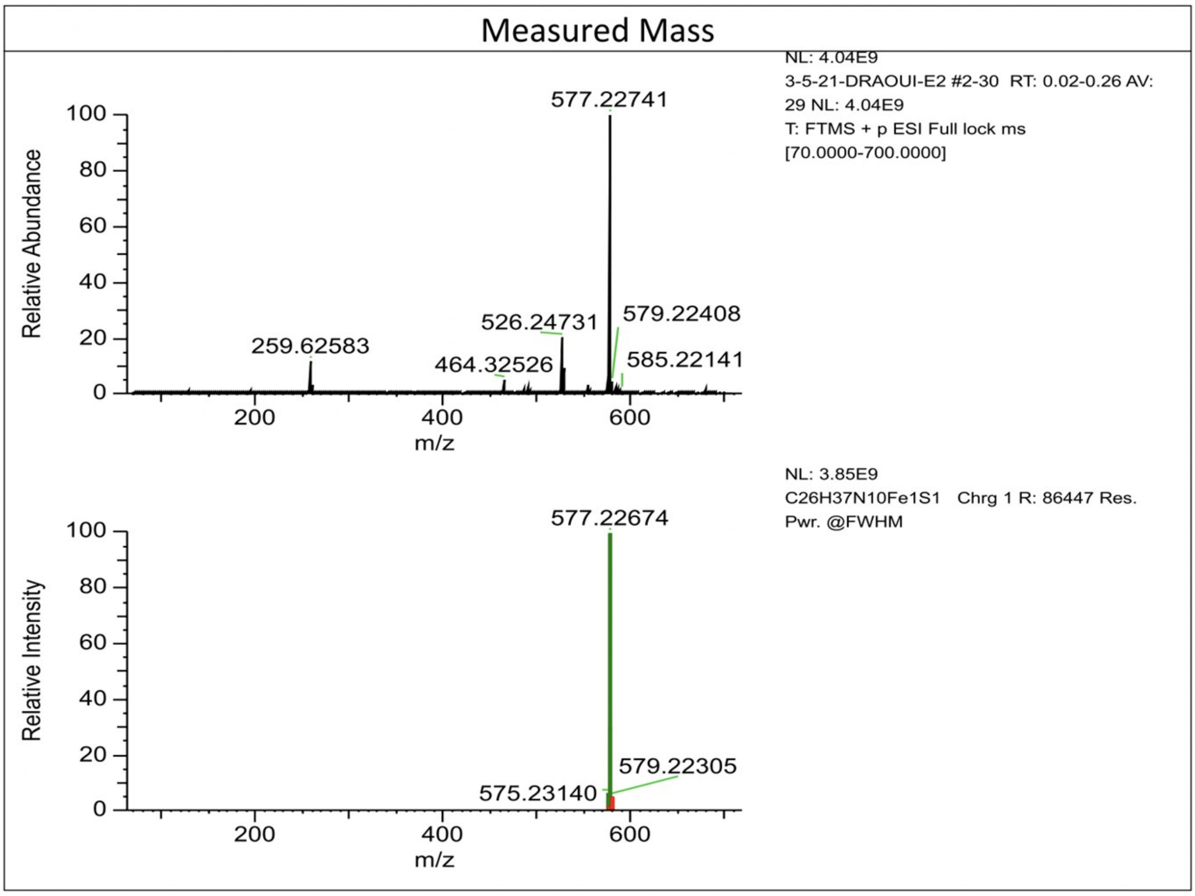

11. Elemental composition of [FeL(NCS)$_2$]:

Peak Mass	Display Formula	MS Cov.[%]	Delta[ppm]	Theo.mass
575.23264	$C_{26}H_{37}N_{10}{}^{54}Fe^{32}S$	100,17	2,15	575.23140

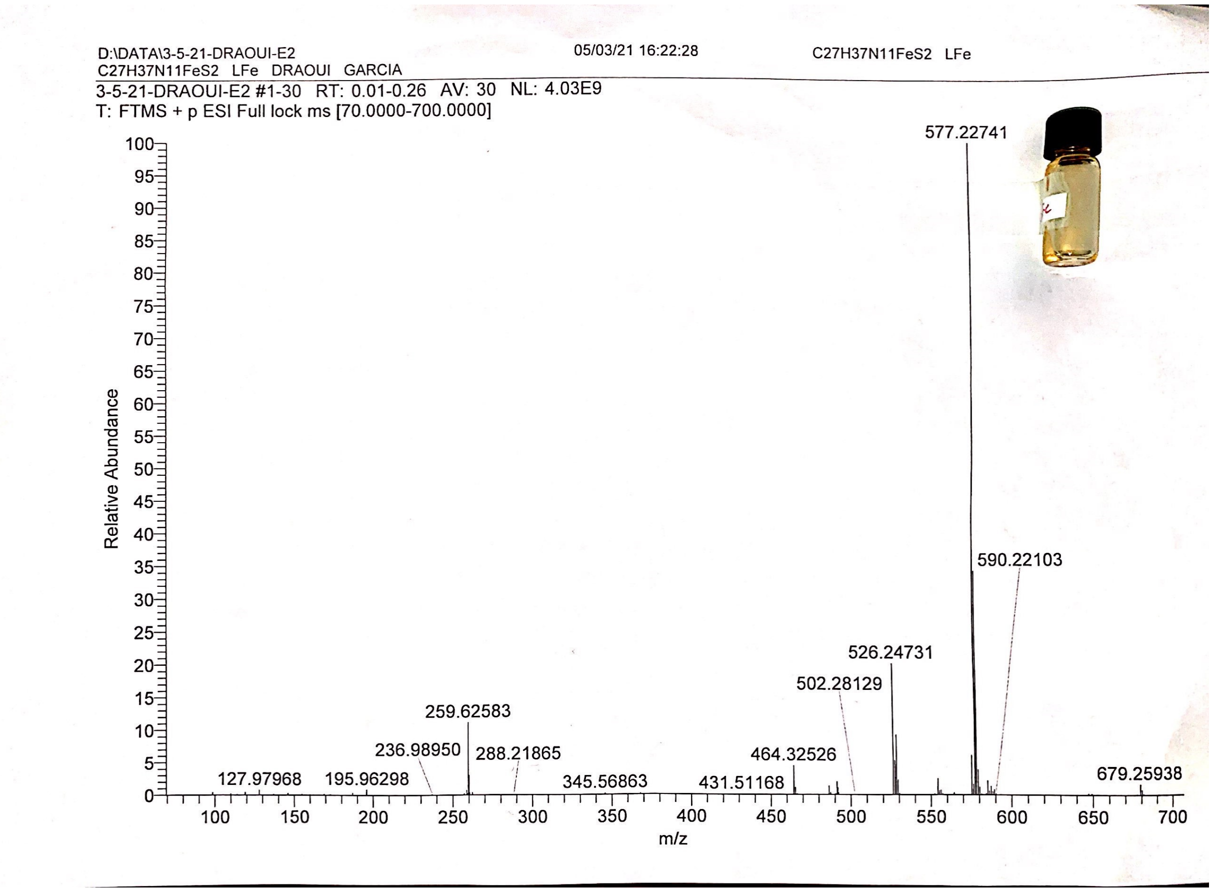
D:\DATA\3-5-21-DRAOUI-E2
05/03/21 16:22:28
C27H37N11FeS2 LFe
C27H37N11FeS2 LFe DRAOUI GARCIA
3-5-21-DRAOUI-E2 #1-30 RT: 0.01-0.26 AV: 30 NL: 4.03E9
T: FTMS + p ESI Full lock ms [70.0000-700.0000]
Relative Abundance
m/z
577.22741
590.22103
526.24731
502.28129
464.32526
431.51168
345.56863
288.21865
259.62583
236.98950
195.96298
127.97968
679.25938

12. HRMS of *CoL(CH$_3$OH)(H$_2$O)***:**

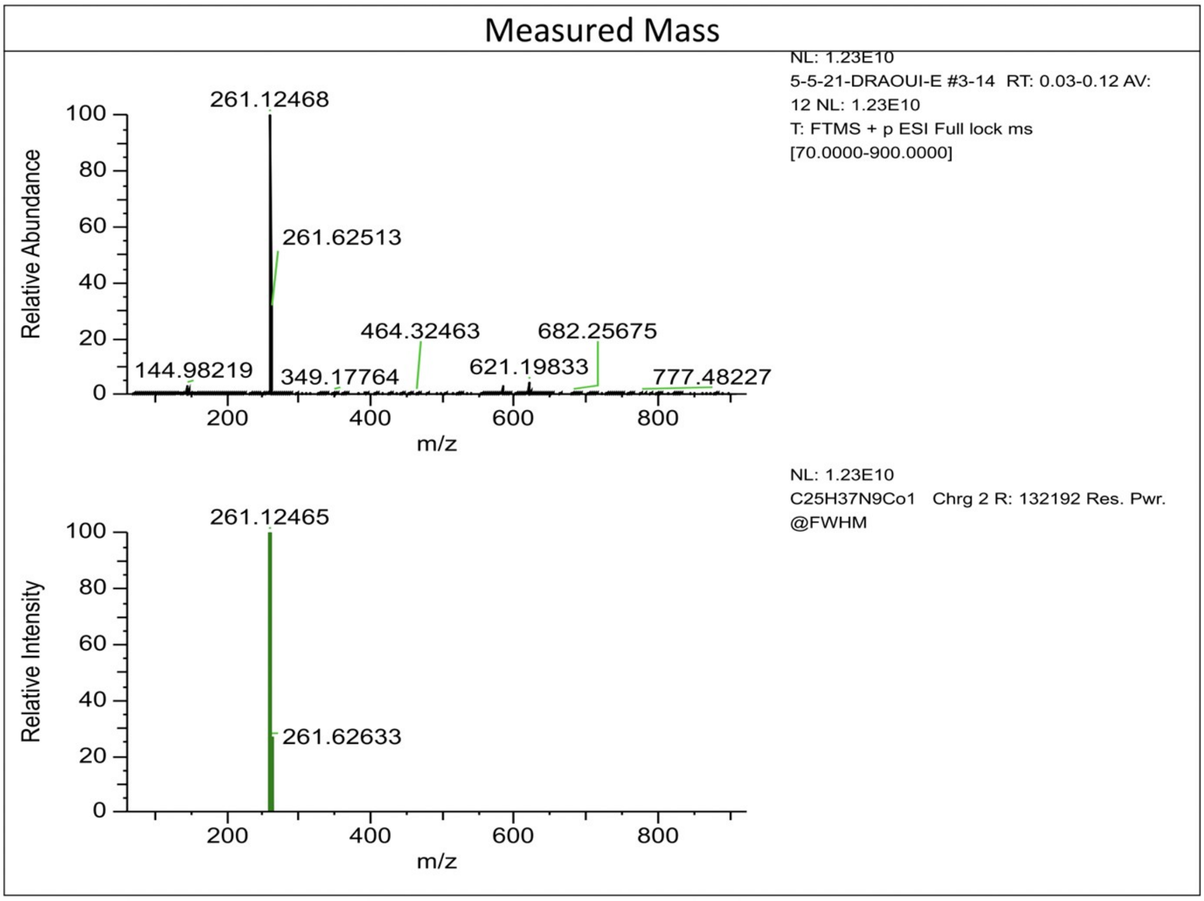

13. Elemental composition of *CoL(CH$_3$OH)(H$_2$O)***:**

Peak Mass	Display Formula	MS Cov.[%]	Delta[ppm]	Theo.mass
261.12468	$C_{25}H_{37}N_9{}^{59}Co$	99.31	0.13	261.12465

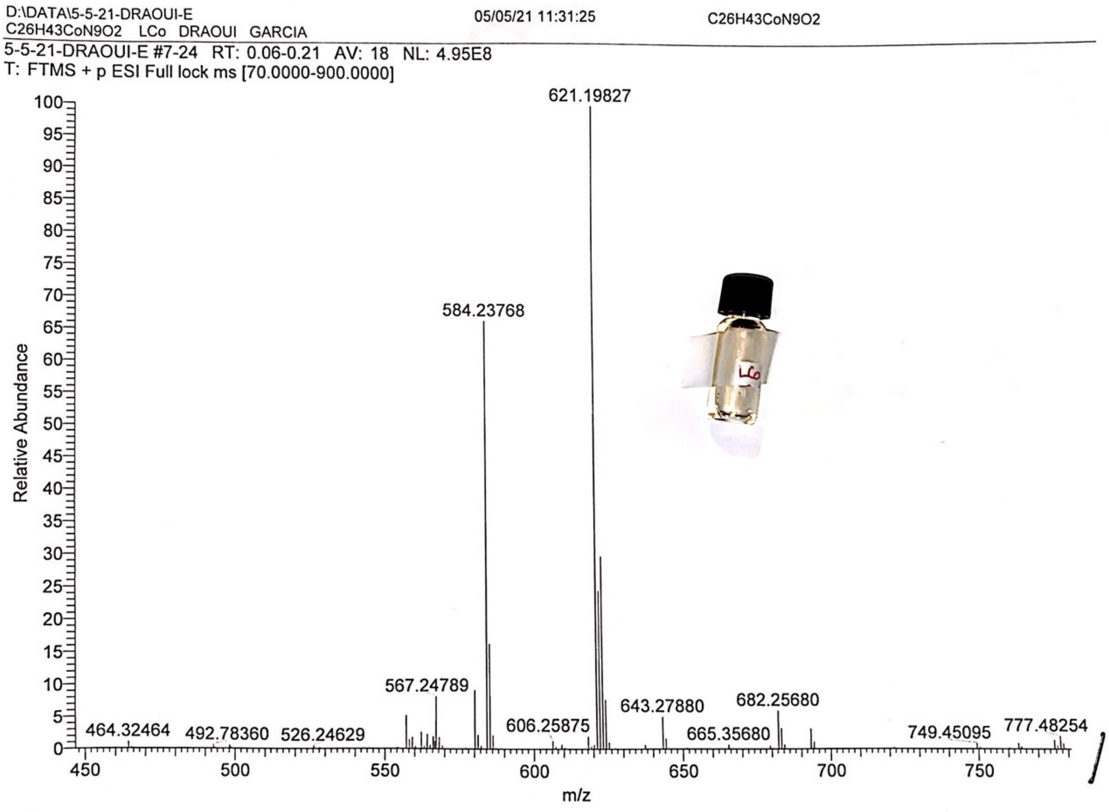
D:\DATA\5-5-21-DRAOUI-E
05/05/21 11:31:25
C26H43CoN9O2
C26H43CoN9O2 LCo DRAOUI GARCIA
5-5-21-DRAOUI-E #7-24 RT: 0.06-0.21 AV: 18 NL: 4.95E8
T: FTMS + p ESI Full lock ms [70.0000-900.0000]
Relative Abundance
m/z
621.19827
584.23768
567.24789
682.25680
643.27880
606.25875
665.35680
464.32464
492.78360
526.24629
749.45095
777.48254
LCo

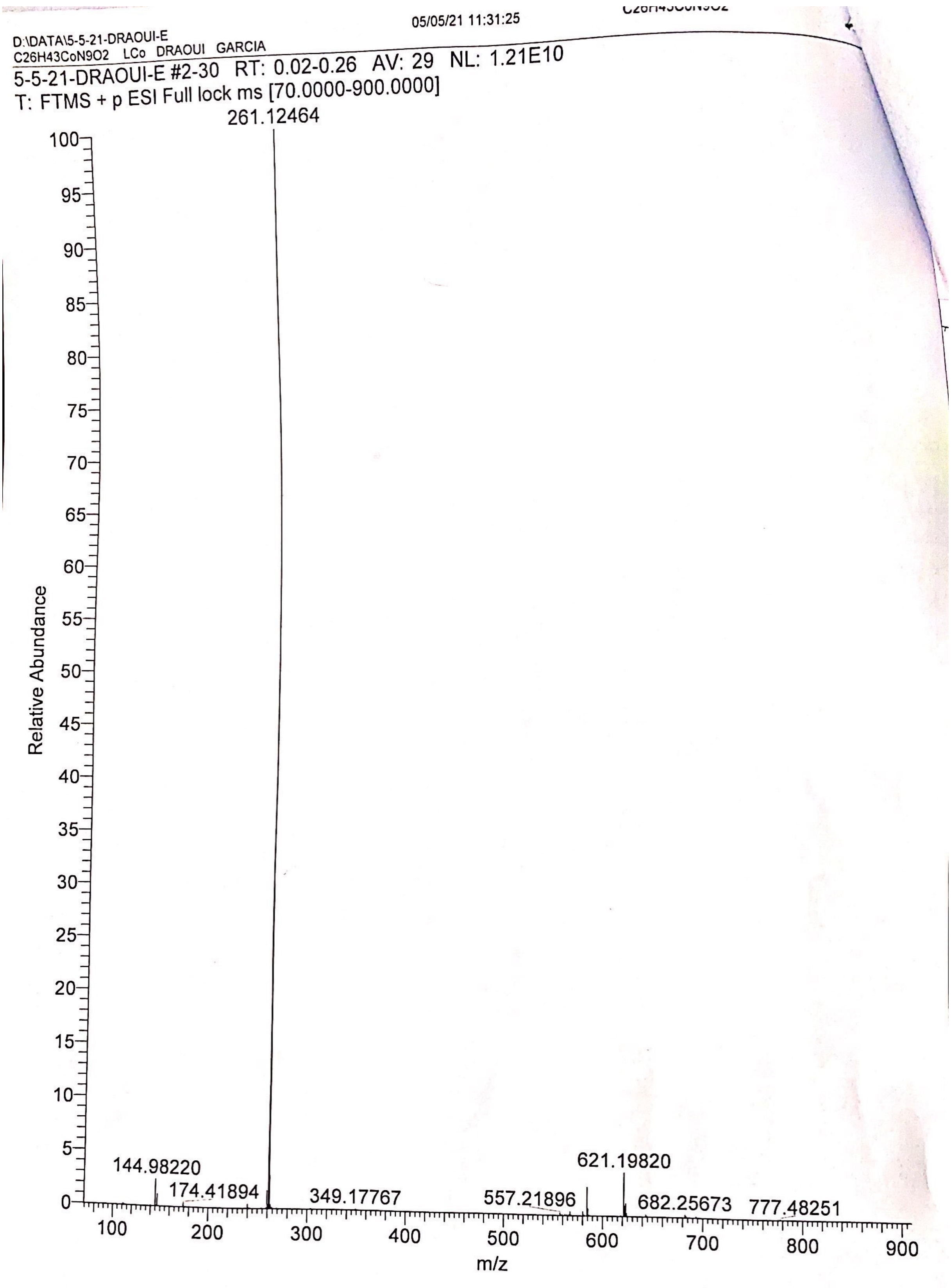

C26H43CoN9O2
D:\DATA\5-5-21-DRAOUI-E
05/05/21 11:31:25
C26H43CoN9O2 LCo DRAOUI GARCIA
5-5-21-DRAOUI-E #2-30 RT: 0.02-0.26 AV: 29 NL: 1.21E10
T: FTMS + p ESI Full lock ms [70.0000-900.0000]
261.12464
144.98220
174.41894
349.17767
557.21896
621.19820
682.25673
777.48251
Relative Abundance
m/z
